# Entanglement and Photon Anti-Bunching in Coupled Non-Degenerate Parametric Oscillators

**DOI:** 10.3390/e23050624

**Published:** 2021-05-17

**Authors:** Yoshitaka Inui, Yoshihisa Yamamoto

**Affiliations:** 1 Physics and Informatics Laboratories, NTT Research Inc., 940 Stewart Dr, Sunnyvale, CA 94085, USA; yoshihisa.yamamoto@ntt-research.com; 2 E. L. Ginzton Laboratory, Stanford University, Stanford, CA 94305, USA

**Keywords:** entanglement, quantum optics, laser

## Abstract

We analytically and numerically show that the Hillery-Zubairy’s entanglement criterion is satisfied both below and above the threshold of coupled non-degenerate optical parametric oscillators (NOPOs) with strong nonlinear gain saturation and dissipative linear coupling. We investigated two cases: for large pump mode dissipation, below-threshold entanglement is possible only when the parametric interaction has an enough detuning among the signal, idler, and pump photon modes. On the other hand, for a large dissipative coupling, below-threshold entanglement is possible even when there is no detuning in the parametric interaction. In both cases, a non-Gaussian state entanglement criterion is satisfied even at the threshold. Recent progress in nano-photonic devices might make it possible to experimentally demonstrate this phase transition in a coherent XY machine with quantum correlations.

## 1. Introduction

Networks of degenerate optical parametric oscillators (DOPOs), called coherent Ising machines (CIMs) [1,2,3,4,5,6,7,8,9,10,11,12,13], have been extensively studied from quantum optics and neural-network perspectives (for a recent review, see Ref. [14]). A DOPO network is constructed with a dissipative (Liouvillian) linear coupling rather than a conservative (Hamiltonian) coupling using either optical delay lines [2,5,6] or homodyne measurement feedback circuits [7,8,12]. The fundamental topics in quantum optics that can be studied with CIMs include Gaussian state entanglement [3,4], the Schrödinger cat state [10], and the entangled cat state [15]. Applications cover a broad spectrum, including spin-glass solvers [16,17], structure-based virtual screening for drug discovery [18], combinatorial optimization [12,19], compressed sensing [20], and fair sampling for deep machine learning [21].

The fundamental quantum resources of CIMs come from the phase-sensitive amplification/deamplification of two quadrature amplitudes in the DOPO [22]. Quantum correlation among DOPOs, evaluated by entanglement and quantum discord, reaches a maximum at the DOPO threshold and decreases below and above the threshold [23]. This quantum resource comes at the cost of limiting the spin-like degrees of freedom for computation and simulation. The in-phase quadrature amplitude (a canonical coordinate of a harmonic oscillator) takes either one of the bi-stable values above the threshold. We can relax this binary constraint using phase-insensitive oscillators such as lasers [24,25,26] or exciton-polariton condensates [27,28], in which the optical or polaritonic field can take a continuous phase above the threshold, so that an XY Hamiltonian (or Kuramoto model) can be naturally implemented instead of the Ising Hamiltonian. We call such a network as a coherent XY machine (CXM) in this paper. However, the bosonic fields in lasers and polariton condensates are thermal statistical mixture states below the threshold, while they approach coherent states far above the threshold [29]. The lack of non-classical states in lasers and polariton condensates seems to exclude the possibility of creating quantum correlations in a CXM based on such classical oscillators.

Non-degenerate optical parametric oscillators (NOPOs) have recently been used to construct a CXM [30]. One of the experimental advantages of an NOPO-based CXM is that we can easily transform a CIM to a CXM by introducing the frequency non-degeneracy between the signal and idler waves without changing the basic structure of the machine. An NOPO is a phase insensitive oscillator with a continuous phase degree of freedom, but its quantum statistical features are unique and distinct from standard lasers and polariton condensates. An NOPO below the threshold has a stronger gain saturation than a standard laser, so that a sub-Poissonian light or even a single photon state may be generated if the system parameters are chosen appropriately. This non-classical behavior below the threshold is analogous to those of a strongly coupled atom-cavity system [31,32] and coherently excited Raman three-level system with a large coupling and detuning [33]. On the other hand, an NOPO above the threshold can produce an amplitude squeezed state with a reduced amplitude fluctuation due to the strong gain saturation and diverging phase fluctuation due to a random walk diffusion process. This non-classical behavior above the threshold is analogous to those of pump-noise-suppressed lasers [34,35,36,37,38,39]. When such non-classical states of light are mixed by dissipative (Liouvillian) coupling, it is expected that the quantum correlations will form among the two NOPOs, just as in the case of two DOPOs in a CIM [3,4,23]. This would be another advantage of NOPO-based CXMs.

In this paper, we investigate the formation of entanglement in a CXM consisting of two NOPOs with a large parametric gain and show that entanglement is achieved below, above, and at the threshold. We use both analytical and numerical methods, although the analytical one is used to calculate the entanglement characteristics only far below and far above the threshold. Our analytical study on above-threshold CXMs, started with *c*-number stochastic differential equations (cSDEs) in the positive-*P* representation [40,41]. The numerical simulations were performed using both the quantum master equation (QME) in the photon number representation, and the wave function Monte Carlo (WFMC) method [42]. The paper is organized as follows. We introduce the model of CXM consisting of two NOPOs in Section 2. In Section 3, we investigate the case of a large dissipation in the pump mode and find that a large detuning of the parametric interaction is required to satisfy the entanglement criterion below the threshold. Next, in Section 4, we consider the case of a large dissipative coupling. We find that, below the threshold, entanglement is obtained even in the absence of detuning in the parametric interaction. Section 5 summarizes the paper. Appendix A shows the Fokker-Planck equation of the positive-*P* quasi-distribution function and derives the below-threshold characteristics of a single NOPO. Appendix B and Appendix C provide subsidiary information on the numerical and theoretical equations. Appendix D and Appendix E provide the detailed derivations of analytical results. Appendix F and Appendix G show the supplementary information about the entanglement criterion and the experimental NOPO [30].

## 2. Model

The density matrix equation of a single $\chi^{\left(2\right)}$ NOPO is written as

(1) $$\begin{array}{c} \frac{\partial \hat{\rho }}{\partial t}=\sum_{a=p,s,i}\gamma_{a}\left(\left[{\hat{a}}_{a},\hat{\rho }{\hat{a}}_{a}^{†}\right]+h.c.\right)+\varepsilon \left[{\hat{a}}_{p}^{†}-{\hat{a}}_{p},\hat{\rho }\right]+\kappa \left[{\hat{a}}_{s}^{†}{\hat{a}}_{i}^{†}{\hat{a}}_{p}e^{-i\Delta t}-{\hat{a}}_{p}^{†}{\hat{a}}_{i}{\hat{a}}_{s}e^{+i\Delta t},\hat{\rho }\right]. \end{array}$$

Here, ${\hat{a}}_{p}$, ${\hat{a}}_{s}$, and ${\hat{a}}_{i}$ are pump, signal, and idler modes, respectively. These three photon modes have cavity frequencies $\omega_{a}\left(a=p,s,i\right)$ and the linear dissipation rates $\gamma_{a}\left(a=p,s,i\right)$. $\Delta =\left(\omega_{p}-\omega_{s}\right)-\omega_{i}$ is the detuning of the parametric interaction. ε is the strength of the coherent excitation of the pump mode. κ is the strength of the $\chi^{\left(2\right)}$ parametric interaction. If the idler mode has a much larger dissipation than the other two photon modes ($\gamma_{i}\gg \gamma_{p},\gamma_{s}$), the quantum master equation can be written as

(2) $$\begin{array}{ccc} {\left.\frac{\partial \hat{\rho }}{\partial t}\right|}_{NOPO} & = & \sum_{a=p,s}\gamma_{a}\left(\left[{\hat{a}}_{a},\hat{\rho }{\hat{a}}_{a}^{†}\right]+h.c.\right)+\varepsilon \left[{\hat{a}}_{p}^{†}-{\hat{a}}_{p},\hat{\rho }\right]\\ & - & iK\left[{\hat{a}}_{p}^{†}{\hat{a}}_{p}{\hat{a}}_{s}^{†}{\hat{a}}_{s},\hat{\rho }\right]+G\left(\left[{\hat{a}}_{s}^{†}{\hat{a}}_{p},\hat{\rho }{\hat{a}}_{p}^{†}{\hat{a}}_{s}\right]+h.c.\right). \end{array}$$

Here *G* is the coefficient of the effective Raman interaction in Refs. [43,44,45], and *K* is the coefficient of the non-degenerate Kerr effect [46]. If Δ=0, the Kerr coefficient is K=0 and the signal mode has a parametric gain $G=\kappa^{2}/\gamma_{i}$. We define the maximum parametric gain as $G_{0}:=\kappa^{2}/\gamma_{i}$. For the detuned parametric interaction, the parametric gain is $G=\frac{\kappa^{2}\gamma_{i}}{\gamma_{i}^{2}+\Delta^{2}}$, and the Kerr coefficient is $K=\frac{\kappa^{2}\Delta }{\gamma_{i}^{2}+\Delta^{2}}$. We use the dimensionless detuning parameter $d=K^{2}/G^{2}$ to represent the normalized detuning. We also introduce the normalized excitation $p=\varepsilon /\varepsilon_{thr}$, where $\varepsilon_{thr}:=\gamma_{p}\sqrt{\gamma_{s}/G}$ is the strength of the excitation at the oscillation threshold.

Let us consider a CXM consisting of two NOPOs (NOPO1 with ${\hat{a}}_{p1},{\hat{a}}_{s1}$ and NOPO2 with ${\hat{a}}_{p2},{\hat{a}}_{s2}$) coupled by a dissipative Liouvillian $L_{c}\hat{\rho }$ for two signal modes [3,4,47,48,49],

(3) $$\frac{\partial \hat{\rho }}{\partial t}={\left.\frac{\partial \hat{\rho }}{\partial t}\right|}_{NOPO1}+{\left.\frac{\partial \hat{\rho }}{\partial t}\right|}_{NOPO2}+L_{c}\hat{\rho },$$

(4) $$L_{c}\hat{\rho }=J\left[{\hat{a}}_{s1}-{\hat{a}}_{s2},\hat{\rho }\left({\hat{a}}_{s1}^{†}-{\hat{a}}_{s2}^{†}\right)\right]+h.c..$$

We will evaluate the entanglement between the two signal modes using one of the entanglement criteria in Ref. [50],

(5) $$HZ1=|\langle {\hat{a}}_{s1}^{†}{\hat{a}}_{s2}\rangle {|}^{2}-\langle {\hat{a}}_{s1}^{†}{\hat{a}}_{s1}{\hat{a}}_{s2}^{†}{\hat{a}}_{s2}\rangle .$$

If this value is larger than zero, the two signal modes are entangled. Above the threshold, we assume the ferromagnetic configuration $\langle {\hat{a}}_{s1}\rangle =\langle {\hat{a}}_{s2}\rangle$ is created by the coupling Liouvillian (4). Using the fluctuation of positive-*P* amplitudes (introduced in Appendix A and Appendix B), the normalized HZ1 is written as HZ1〈a^s†a^s〉∼−2〈Δαs1†Δαs1〉−2Re〈Δαs1Δαs2〉. Here, we assumed $\langle \Delta \alpha_{s1}^{†}\Delta \alpha_{s1}\rangle =\langle \Delta \alpha_{s2}^{†}\Delta \alpha_{s2}\rangle$ and that $\langle {\hat{a}}_{s1}\rangle$ is real. Introducing the canonical coordinates and canonical momenta as $\Delta X_{si}=\frac{\Delta \alpha_{si}+\Delta \alpha_{si}^{†}}{\sqrt{2}}$ and $\Delta P_{si}=\frac{\Delta \alpha_{si}-\Delta \alpha_{si}^{†}}{\sqrt{2}i}\left(i=1,2\right)$, this normalized entanglement criterion is written as

(6) $$\frac{HZ1}{\langle {\hat{a}}_{s}^{†}{\hat{a}}_{s}\rangle }=-\langle \Delta X_{s1}^{2}\rangle -\langle \Delta X_{s1}\Delta X_{s2}\rangle -\langle \Delta P_{s1}^{2}\rangle +\langle \Delta P_{s1}\Delta P_{s2}\rangle .$$

This criterion is equivalent to the Duan’s sufficient condition for entanglement (Theorem 1 of Ref. [51]). Moreover, HZ1 criterion can detect an entanglement in a non-Gaussian state, for example, entanglement of a superposition of single photon states $|\psi \rangle =\frac{1}{\sqrt{2}}(|0,1\rangle +\left|1,0\rangle \right)$ [52]. This criterion seems useful to detect non-Gaussian entanglement in the below-threshold CXM, since the similar model [33] realizes a single photon state.

## 3. Large Pump Dissipation Limit

### 3.1. Quantum Master Equation after the Elimination of the Pump Mode

In this section, we consider the large pump mode dissipation limit, where $G,K,\gamma_{p}\;\gg \;\gamma_{s},$ *J*. In this limit, the linear loss of the pump mode, due to the dissipation into the reservoir and spontaneous emission into the signal mode, is much larger than the linear loss of the signal mode. We will use the expansion of the density matrix with the complex-*P* representation [53,54] for the pump mode and photon number state representation for the signal mode:

(7) $$\hat{\rho }=\sum_{N_{s},N_{s}^{\prime }}\int P_{N_{s},N_{s}^{\prime }}\left(\alpha_{p},\alpha_{p}^{†}\right)\frac{|\alpha_{p}\rangle \langle \alpha_{p}^{†*}|}{\langle \alpha_{p}^{†*}|\alpha_{p}\rangle }⊗|N_{s}\rangle \langle N_{s}^{\prime }|d\alpha_{p}d\alpha_{p}^{†}.$$

Substituting this expansion into Equation (2), we can obtain a time development equation for $P_{N_{s},N_{s}^{\prime }}\left(\alpha_{p},\alpha_{p}^{†}\right)$.

(8) $$\begin{array}{ccc} \frac{\partial P_{N_{s},N_{s}^{\prime }}}{\partial t} & = & \gamma_{s}\left[2\sqrt{\left(1+N_{s}\right)\left(1+N_{s}^{\prime }\right)}P_{N_{s}+1,N_{s}^{\prime }+1}-\left(N_{s}+N_{s}^{\prime }\right)P_{N_{s},N_{s}^{\prime }}\right]-\varepsilon \frac{\partial P_{N_{s},N_{s}^{\prime }}}{\partial \alpha_{p}}-\varepsilon \frac{\partial P_{N_{s},N_{s}^{\prime }}}{\partial \alpha_{p}^{†}}\\ & + & \frac{\partial }{\partial \alpha_{p}}\left(\gamma_{p}+G\left(1+N_{s}\right)\right)\alpha_{p}P_{N_{s},N_{s}^{\prime }}+iK\frac{\partial }{\partial \alpha_{p}}N_{s}\alpha_{p}P_{N_{s},N_{s}^{\prime }}\\ & + & \frac{\partial }{\partial \alpha_{p}^{†}}\left(\gamma_{p}+G\left(1+N_{s}^{\prime }\right)\right)\alpha_{p}^{†}P_{N_{s},N_{s}^{\prime }}-iK\frac{\partial }{\partial \alpha_{p}^{†}}N_{s}^{\prime }\alpha_{p}^{†}P_{N_{s},N_{s}^{\prime }}\\ & - & iK\alpha_{p}^{†}\alpha_{p}\left(N_{s}-N_{s}^{\prime }\right)P_{N_{s},N_{s}^{\prime }}+G\alpha_{p}^{†}\alpha_{p}\left[2\sqrt{N_{s}N_{s}^{\prime }}P_{N_{s}-1,N_{s}^{\prime }-1}-\left(2+N_{s}+N_{s}^{\prime }\right)P_{N_{s},N_{s}^{\prime }}\right]. \end{array}$$

Due to the linear dissipation of the signal mode ($\gamma_{s}$), components with photon number indices $\left(N_{s},N_{s}^{\prime }\right)$ are excited by components with larger photon number indices $\left(N_{s}+1,N_{s}^{\prime }+1\right)$. The parametric gain ($G\alpha_{p}^{†}\alpha_{p}$) introduces a contribution from components with smaller photon number indices $\left(N_{s}-1,N_{s}^{\prime }-1\right)$. Other processes do not affect the photon number indices. Although the equation has drift terms for the pump amplitudes $\left(\alpha_{p},\alpha_{p}^{†}\right)$, the signal photon number indices do not change when components are derived using $\alpha_{p}$ or $\alpha_{p}^{†}$. When $\gamma_{p}+G$ is sufficiently large, the complex-*P* amplitude ($\alpha_{p}$) at photon number indices ($N_{s}$, $N_{s}^{\prime }$) rapidly converge to the steady-state value of the time-development equation $\frac{d\alpha_{p}}{dt}=-\left(\gamma_{p}+G\left(1+N_{s}\right)+iKN_{s}\right)\alpha_{p}+\varepsilon$. We can eliminate the pump mode amplitudes by writing $P_{N_{s},N_{s}^{\prime }}\left(\alpha_{p},\alpha_{p}^{†}\right)=\rho_{N_{s},N_{s}^{\prime }}\delta \left(\alpha_{p}-\frac{\varepsilon }{\gamma_{p}+G\left(1+N_{s}\right)+iKN_{s}}\right)\delta \left(\alpha_{p}^{†}-\frac{\varepsilon }{\gamma_{p}+G\left(1+N_{s}^{\prime }\right)-iKN_{s}^{\prime }}\right)$, and integrating the complex-*P* amplitudes by $\int d\alpha_{p}d\alpha_{p}^{†}$. The density matrix components of the signal mode $\rho_{N_{s},N_{s}^{\prime }}=\langle N_{s}|\hat{\rho }|N_{s}^{\prime }\rangle$ are,

(9) $$\begin{array}{ccc} \frac{\partial \rho_{N_{s},N_{s}^{\prime }}}{\partial t} & = & 2\gamma_{s}\sqrt{\left(N_{s}+1\right)\left(N_{s}^{\prime }+1\right)}\rho_{N_{s}+1,N_{s}^{\prime }+1}-\gamma_{s}\left(N_{s}+N_{s}^{\prime }\right)\rho_{N_{s},N_{s}^{\prime }}\\ & - & iK_{e}\left(N_{s},N_{s}^{\prime }\right)\left(N_{s}-N_{s}^{\prime }\right)\rho_{N_{s},N_{s}^{\prime }}\\ & + & 2G_{e}\left(N_{s}-1,N_{s}^{\prime }-1\right)\sqrt{N_{s}N_{s}^{\prime }}\rho_{N_{s}-1,N_{s}^{\prime }-1}-G_{e}\left(N_{s},N_{s}^{\prime }\right)\left(2+N_{s}+N_{s}^{\prime }\right)\rho_{N_{s},N_{s}^{\prime }}. \end{array}$$

Here $G_{e}\left(N,N^{\prime }\right)=\frac{G\varepsilon^{2}}{\left[\gamma_{p}+G\left(1+N\right)+iKN\right]\left[\gamma_{p}+G\left(1+N^{\prime }\right)-iKN^{\prime }\right]}$, and $K_{e}\left(N,N^{\prime }\right)=\frac{K\varepsilon^{2}}{\left[\gamma_{p}+G\left(1+N\right)+iKN\right]\left[\gamma_{p}+G\left(1+N^{\prime }\right)-iKN^{\prime }\right]}$. The denominators of these terms have higher dependence on the signal photon number than in Scully–Lamb’s theory [55]. In regard to the CXM consisting of two NOPOs, we can omit the subscript for the signal mode *s*, after eliminating the pump mode. The density matrix components of the two signal modes $\rho_{N_{1},N_{2},N_{1}^{\prime },N_{2}^{\prime }}=\langle N_{1},N_{2}|\hat{\rho }|N_{1}^{\prime },N_{2}^{\prime }\rangle$ develop as,

(10) $$\begin{array}{ccc} & & \frac{\partial \rho_{N_{1},N_{2},N_{1}^{\prime },N_{2}^{\prime }}}{\partial t}=\left(\gamma_{s}+J\right)\left[2\sqrt{\left(N_{1}+1\right)\left(N_{1}^{\prime }+1\right)}\rho_{N_{1}+1,N_{2},N_{1}^{\prime }+1,N_{2}^{\prime }}-\left(N_{1}+N_{1}^{\prime }\right)\rho_{N_{1},N_{2},N_{1}^{\prime },N_{2}^{\prime }}\right]\\ & + & 2G_{e}\left(N_{1}-1,N_{1}^{\prime }-1\right)\sqrt{N_{1}N_{1}^{\prime }}\rho_{N_{1}-1,N_{2},N_{1}^{\prime }-1,N_{2}^{\prime }}-G_{e}\left(N_{1},N_{1}^{\prime }\right)\left(2+N_{1}+N_{1}^{\prime }\right)\rho_{N_{1},N_{2},N_{1}^{\prime },N_{2}^{\prime }}\\ & + & \left(\gamma_{s}+J\right)\left[2\sqrt{\left(N_{2}+1\right)\left(N_{2}^{\prime }+1\right)}\rho_{N_{1},N_{2}+1,N_{1}^{\prime },N_{2}^{\prime }+1}-\left(N_{2}+N_{2}^{\prime }\right)\rho_{N_{1},N_{2},N_{1}^{\prime },N_{2}^{\prime }}\right]\\ & + & 2G_{e}\left(N_{2}-1,N_{2}^{\prime }-1\right)\sqrt{N_{2}N_{2}^{\prime }}\rho_{N_{1},N_{2}-1,N_{1}^{\prime },N_{2}^{\prime }-1}-G_{e}\left(N_{2},N_{2}^{\prime }\right)\left(2+N_{2}+N_{2}^{\prime }\right)\rho_{N_{1},N_{2},N_{1}^{\prime },N_{2}^{\prime }}\\ & - & iK_{e}\left(N_{1},N_{1}^{\prime }\right)\left(N_{1}-N_{1}^{\prime }\right)\rho_{N_{1},N_{2},N_{1}^{\prime },N_{2}^{\prime }}-iK_{e}\left(N_{2},N_{2}^{\prime }\right)\left(N_{2}-N_{2}^{\prime }\right)\rho_{N_{1},N_{2},N_{1}^{\prime },N_{2}^{\prime }}\\ & - & J[2\sqrt{\left(N_{1}+1\right)\left(N_{2}^{\prime }+1\right)}\rho_{N_{1}+1,N_{2},N_{1}^{\prime },N_{2}^{\prime }+1}+2\sqrt{\left(N_{2}+1\right)\left(N_{1}^{\prime }+1\right)}\rho_{N_{1},N_{2}+1,N_{1}^{\prime }+1,N_{2}^{\prime }}\\ & - & \sqrt{\left(N_{1}+1\right)N_{2}}\rho_{N_{1}+1,N_{2}-1,N_{1}^{\prime },N_{2}^{\prime }}-\sqrt{N_{1}^{\prime }\left(N_{2}^{\prime }+1\right)}\rho_{N_{1},N_{2},N_{1}^{\prime }-1,N_{2}^{\prime }+1}\\ & - & \sqrt{\left(N_{2}+1\right)N_{1}}\rho_{N_{1}-1,N_{2}+1,N_{1}^{\prime },N_{2}^{\prime }}-\sqrt{N_{2}^{\prime }\left(N_{1}^{\prime }+1\right)}\rho_{N_{1},N_{2},N_{1}^{\prime }+1,N_{2}^{\prime }-1}]. \end{array}$$

### 3.2. Far-Below-Threshold Entanglement

From the above Equation (10), we will analytically derive the photon anti-bunching and entanglement characteristics far below the threshold (p≪1). In this limit, $G_{e}\left(N_{i},N_{i}^{\prime }\right)$ and $K_{e}\left(N_{i},N_{i}^{\prime }\right)\left(i=1,2\right)$ are of order $O(p^{2})$. Therefore, these contributions to $\rho_{N_{1},N_{2},N_{1}^{\prime },N_{2}^{\prime }}$ on the right hand side of Equation (10) are much smaller than those of $\gamma_{s}$ and *J*. However, the $G_{e}\left(N_{i}-1,N_{i}^{\prime }-1\right)\left(i=1,2\right)$ on the right hand side are not negligible. Although the $K_{e}\left(N_{i},N_{i}^{\prime }\right)\left(i=1,2\right)$ on the right hand side do not contribute for small *p*, the non-degenerate Kerr coefficient *K* in the denominators of $G_{e}\left(N_{i}-1,N_{i}^{\prime }-1\right)\left(i=1,2\right)$ contributes to the characteristics far below the threshold. The signal photon number of the CXM is obtained from the equations of the two density-matrix components, $\rho_{10,10}$, and $\rho_{10,01}$.

(11) $$\frac{\partial \rho_{10,10}}{\partial t}=-2\left(\gamma_{s}+J\right)\rho_{10,10}+2J\rho_{10,01}+2G_{e}\left(0,0\right)\rho_{00,00},$$

(12) $$\frac{\partial \rho_{10,01}}{\partial t}=-2\left(\gamma_{s}+J\right)\rho_{10,01}+2J\rho_{10,10}.$$

From these equations, the mean signal photon number is $\rho_{10,10}/\rho_{00,00}$. In the steady-state, this becomes

(13) $$\langle {\hat{a}}_{s1}^{†}{\hat{a}}_{s1}\rangle =\frac{\gamma_{s}+J}{\gamma_{s}+2J}\frac{\gamma_{p}^{2}}{{\left(\gamma_{p}+G\right)}^{2}}p^{2}.$$

For a weak nonlinear gain saturation ($G\ll \gamma_{p}$), this value reaches one at p∼1. However, in general, $p∼1+G/\gamma_{p}$ is required for achieving $\langle {\hat{a}}_{s1}^{†}{\hat{a}}_{s1}\rangle =1$, where stimulated emission becomes dominant over spontaneous emission. $\rho_{10,01}/\rho_{00,00}$ provides an amplitude correlation function between the two signal modes,

(14) $$\langle {\hat{a}}_{s1}^{†}{\hat{a}}_{s2}\rangle =\frac{J}{\gamma_{s}+2J}\frac{\gamma_{p}^{2}}{{\left(\gamma_{p}+G\right)}^{2}}p^{2}.$$

Next, we calculate the values of order $O(p^{4})$ from the equations of four components, $\rho_{20,20}$, $\rho_{11,11}$, $\rho_{20,02}$ and $\rho_{20,11}$.

(15) ∂ρ20,20∂t=−4(γs+J)ρ20,20+22JReρ20,11+4Ge(1,1)ρ10,10,

(16) ∂ρ11,11∂t=−4(γs+J)ρ11,11+42JReρ20,11+4Ge(0,0)ρ10,10,

(17) ∂ρ20,02∂t=−4(γs+J)ρ20,02+22JReρ20,11,

(18) $$\frac{\partial \rho_{20,11}}{\partial t}=-4\left(\gamma_{s}+J\right)\rho_{20,11}+\sqrt{2}J\left(\rho_{20,02}+\rho_{11,11}+\rho_{20,20}\right)+2\sqrt{2}G_{e}\left(1,0\right)\rho_{10,01}.$$

The second-order correlation function $g_{s}^{\left(2\right)}\left(0\right)=\frac{\langle {\hat{a}}_{s1}^{†2}{\hat{a}}_{s1}^{2}\rangle }{{\langle {\hat{a}}_{s1}^{†}{\hat{a}}_{s1}\rangle }^{2}}$ is obtained as $g_{s}^{\left(2\right)}\left(0\right)=2\rho_{20,20}\rho_{00,00}/\rho_{10,10}^{2}$. Its steady-state value is

(19) $$g_{s}^{\left(2\right)}\left(0\right)=\frac{4\gamma_{s}\left(\gamma_{s}+2J\right){\left(\gamma_{p}+G\right)}^{2}+J^{2}\left[K^{2}+{\left(2\gamma_{p}+3G\right)}^{2}\right]}{2{\left(\gamma_{s}+J\right)}^{2}\left[{\left(\gamma_{p}+2G\right)}^{2}+K^{2}\right]}.$$

When the parametric coefficients are sufficiently small ($G,K\ll \gamma_{p}$), the signal modes are in blackbody radiation states with $g_{s}^{\left(2\right)}\left(0\right)=2$ [29]. In the case of a large parametric gain (G,K), however, the signal modes can have non-classical (photon anti-bunching) states with $g_{s}^{\left(2\right)}\left(0\right)<1$. In the single NOPO limit (J→0), $g_{s}^{\left(2\right)}\left(0\right)=\frac{2{\left(\gamma_{p}+G\right)}^{2}}{{\left(\gamma_{p}+2G\right)}^{2}+K^{2}}$ is obtained. This is identical to the $\gamma_{s}\to 0$ limit of $g_{s}^{\left(2\right)}\left(0\right)$ for a single NOPO, which is derived in Appendix A and shown in Equation (A15). This expression converges to a completely anti-bunching state $g_{s}^{\left(2\right)}\left(0\right)=0$ in the K→∞ limit [33]. In the $\gamma_{s}\ll J$ limit, $g_{s}^{\left(2\right)}\left(0\right)$ is $\frac{K^{2}+{\left(2\gamma_{p}+3G\right)}^{2}}{2[K^{2}+{\left(\gamma_{p}+2G\right)}^{2}]}$, and it converges to 0.5 in the large-*K* limit. This is larger than the minimum value for a single NOPO ($g_{s}^{\left(2\right)}\left(0\right)=0$). Hillery-Zubairy’s entanglement criterion (Equation (5)) for the two signal modes is obtained as $HZ1/{\langle {\hat{a}}_{s}^{†}{\hat{a}}_{s}\rangle }^{2}=\rho_{10,01}^{2}/\rho_{10,10}^{2}-\rho_{11,11}\rho_{00,00}/\rho_{10,10}^{2}$, i.e.,

(20) $$\frac{HZ1}{{\langle {\hat{a}}_{s}^{†}{\hat{a}}_{s}\rangle }^{2}}=\frac{J^{2}\left[K^{2}-\left(2\gamma_{p}^{2}+4\gamma_{p}G+G^{2}\right)\right]}{2{\left(\gamma_{s}+J\right)}^{2}\left[{\left(\gamma_{p}+2G\right)}^{2}+K^{2}\right]}-\frac{\gamma_{s}\left(\gamma_{s}+2J\right)}{{\left(\gamma_{s}+J\right)}^{2}}.$$

If this value is larger than zero, the two NOPOs are entangled far below the threshold. From the equation, HZ1 is always negative if K≤G. To achieve entanglement, the detuning of the parametric interaction must be larger than the idler linewidth (d>1). For below-threshold entanglement, a small $\gamma_{p}/G$ ratio and large *J* are preferred.

**Figure 1:**
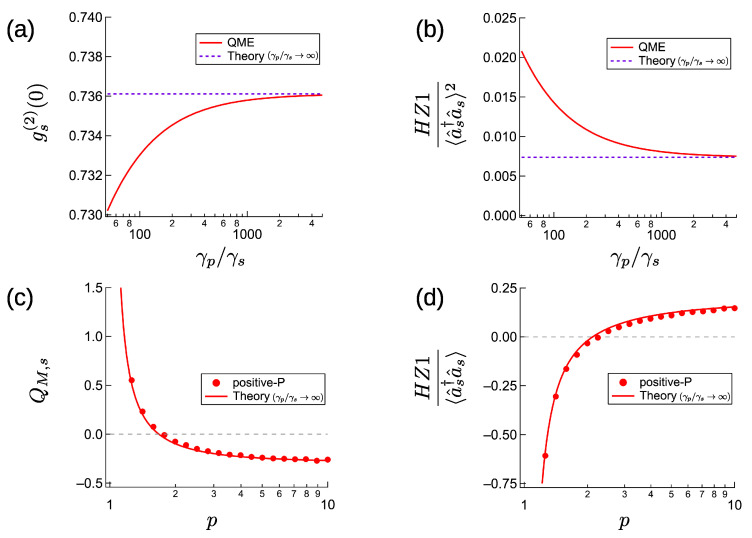
Below- and above-threshold characteristics of CXM with large pump dissipation for d=5, $J/\gamma_{s}=12$. (**a**,**b**) Comparison of far-below-threshold theory ($\gamma_{p}/\gamma_{s}\to \infty$) and quantum master equation with p=0.01 for $G/\gamma_{p}=8$. (**c**,**d**) Comparison of far-above-threshold theory ($\gamma_{p}/\gamma_{s}\to \infty$) and positive-*P* numerical calculation with $\gamma_{p}/\gamma_{s}=50$ and $G/\gamma_{s}=10^{-7}$.

The following is an example of a parameter set for below-threshold entanglement, $J/\gamma_{s}=12$, $G=8\gamma_{p}$, and d=5. These parameters give $g_{s}^{\left(2\right)}\left(0\right)∼0.7361$ and $HZ1/{\langle {\hat{a}}_{s}^{†}{\hat{a}}_{s}\rangle }^{2}∼0.0074$. Therefore, the two NOPOs have anti-bunching states and are entangled. Figure 1a,b compares the analytically and numerically calculated $g_{s}^{\left(2\right)}\left(0\right)$ and $HZ1/{\langle {\hat{a}}_{s}^{†}{\hat{a}}_{s}\rangle }^{2}$. The numerical results are obtained by time developing the quantum master Equation (3) from the vacuum state $\hat{\rho }=\left|0\rangle \langle 0\right|$ in accordance with the pump schedule $p\left(t\right)=0.01\min\left(1,\sqrt{t\gamma_{s}/2}\right)$. The values at $t\gamma_{s}=10$ are used as the approximate steady-state results for p=0.01, and are plotted for various $\gamma_{p}/\gamma_{s}$ ratio. When $\gamma_{p}/\gamma_{s}$ becomes larger than $J/\gamma_{s}=12$ by two orders of magnitude, $g_{s}^{\left(2\right)}\left(0\right)$ and the below-threshold entanglement criterion converge to the analytical results that assume infinite $\gamma_{p}/\gamma_{s}$.

### 3.3. Far-Above-Threshold Entanglement

Next, we analytically derive the above-threshold characteristics of the CXM by assuming a large pump dissipation $\gamma_{p}\gg \gamma_{s}$ and small parametric gain $G\ll \gamma_{s}$. We derive an analytical expression for the above-threshold fluctuations from the equations of positive-*P* representation [40,41]. For a single NOPO, the Fokker-Planck equation of the positive-*P* quasi-distribution function $P(\alpha_{p},\alpha_{p}^{†},\alpha_{s},\alpha_{s}^{†})$ is derived in Appendix A. Here, $\alpha_{p},\alpha_{p}^{†}\left(\alpha_{s},\alpha_{s}^{†}\right)$ are positive-*P* amplitudes for the pump (signal) mode. Using the Ito rule, the equivalent *c*-number stochastic differential equations are expressed as:

(21) $$\frac{d\alpha_{p}}{dt}=-\left(\gamma_{p}+G\right)\alpha_{p}+\varepsilon -\left(G+iK\right)\alpha_{s}^{†}\alpha_{s}\alpha_{p}-\sqrt{\frac{G+iK}{2}}\alpha_{p}\xi_{C},$$

(22) $$\frac{d\alpha_{p}^{†}}{dt}=-\left(\gamma_{p}+G\right)\alpha_{p}^{†}+\varepsilon -\left(G-iK\right)\alpha_{s}^{†}\alpha_{s}\alpha_{p}^{†}-\sqrt{\frac{G-iK}{2}}\alpha_{p}^{†}\xi_{C}^{†},$$

(23) $$\frac{d\alpha_{s}}{dt}=-\gamma_{s}\alpha_{s}+\left(G-iK\right)\alpha_{p}^{†}\alpha_{p}\alpha_{s}+\sqrt{\frac{G+iK}{2}}\alpha_{s}\xi_{C}^{*}+\sqrt{G}\alpha_{p}\xi_{C}^{‡},$$

(24) $$\frac{d\alpha_{s}^{†}}{dt}=-\gamma_{s}\alpha_{s}^{†}+\left(G+iK\right)\alpha_{p}^{†}\alpha_{p}\alpha_{s}^{†}+\sqrt{\frac{G-iK}{2}}\alpha_{s}^{†}\xi_{C}^{†*}+\sqrt{G}\alpha_{p}^{†}\xi_{C}^{‡*}.$$

Here $\xi_{C}$, $\xi_{C}^{†}$ and $\xi_{C}^{‡}$ are independent complex Gaussian random numbers satisfying correlation functions, such as $\langle \xi_{C}^{*}\left(t\right)\xi_{C}\left(t^{\prime }\right)\rangle =2\delta \left(t-t^{\prime }\right)$. $\xi_{C}^{‡}$ reflects random spontaneous emission of signal photons. $\xi_{C}$ and $\xi_{C}^{†}$ contribute to the correlation between the pump and signal amplitudes. From Equations (23) and (24), the above-threshold pump photon number is identical to that of an NOPO without a Kerr term *K* [39]:

(25) $$\langle \alpha_{p}^{†}\alpha_{p}\rangle =\gamma_{s}/G.$$

The above-threshold signal photon number is obtained from $\gamma_{s}/G∼{\left|\langle \alpha_{p}\rangle \right|}^{2}$ and

(26) $$\langle \alpha_{p}\rangle ∼\frac{\varepsilon }{\gamma_{p}+\left(G+iK\right)\langle \alpha_{s}^{†}\alpha_{s}\rangle },$$

which is the steady-state mean of Equation (21), $\frac{d\langle \alpha_{p}\rangle }{dt}∼\varepsilon -\gamma_{p}\langle \alpha_{p}\rangle -\left(G+iK\right)\langle \alpha_{p}\rangle \langle \alpha_{s}^{†}\alpha_{s}\rangle$. Here, we have assumed that $G\ll \gamma_{p}$ and $\langle \alpha_{p}\alpha_{s}^{†}\alpha_{s}\rangle ∼\langle \alpha_{p}\rangle \langle \alpha_{s}^{†}\alpha_{s}\rangle$. Using $d=K^{2}/G^{2}$ and $\varepsilon =\gamma_{p}p\sqrt{\gamma_{s}/G}$, the steady-state signal photon number is obtained as

(27) $$\langle \alpha_{s}^{†}\alpha_{s}\rangle =\frac{\gamma_{p}}{G}\frac{\pi -1}{1+d}.$$

Here, $\pi =\sqrt{p^{2}\left(1+d\right)-d}$ is the normalized excitation modified by the detuning parameter $d=K^{2}/G^{2}$ in the parametric interaction. The steady-state pump amplitude is written as

(28) $$\langle \alpha_{p}\rangle =\sqrt{\frac{\gamma_{s}}{G}}p\frac{1-i\sqrt{d}}{\pi -i\sqrt{d}}.$$

By introducing the phase factor

(29) $$\tan\phi =\frac{\sqrt{d}\left(\pi -1\right)}{\pi +d},$$

the mean pump amplitude becomes $\langle \alpha_{p}\rangle =\sqrt{\frac{\gamma_{s}}{G}}e^{-i\phi }$.

Now let us introduce the small amplitude fluctuations in the pump and signal modes, $\Delta \alpha_{p}$ and $\Delta \alpha_{s}$. The pump amplitude fluctuation is defined as the fluctuation after removing the phase factor denoted by ϕ.

(30) $$\alpha_{p}∼e^{-i\phi }\left(\sqrt{\frac{\gamma_{s}}{G}}+\Delta \alpha_{p}\right).$$

The mean signal amplitude rotates with the frequency $K\langle \alpha_{p}^{†}\alpha_{p}\rangle ∼\sqrt{d}\gamma_{s}$ due to Kerr-nonlinearity. The signal amplitude fluctuation is defined as the fluctuation after removing this time-dependent phase factor,

(31) $$\alpha_{s}∼e^{-i\sqrt{d}\gamma_{s}t}\left(\sqrt{\frac{\gamma_{p}}{G}\frac{\pi -1}{1+d}}+\Delta \alpha_{s}\right).$$

These phase factors do not affect the products, $\alpha_{p}^{†}\alpha_{p}$ and $\alpha_{s}^{†}\alpha_{s}$, appearing in the positive-*P* equations. The equations for small amplitude fluctuations are

(32) $$\frac{d\Delta \alpha_{p}}{dt}=-rp^{2}\frac{1+i\sqrt{d}}{\pi +i\sqrt{d}}\Delta \alpha_{p}-\left(1+i\sqrt{d}\right)\sqrt{\frac{r(\pi -1)}{1+d}}\left(\Delta \alpha_{s}^{†}+\Delta \alpha_{s}\right)-\sqrt{\frac{1+i\sqrt{d}}{2}}\xi_{C},$$

(33) $$\frac{d\Delta \alpha_{p}^{†}}{dt}=-rp^{2}\frac{1-i\sqrt{d}}{\pi -i\sqrt{d}}\Delta \alpha_{p}^{†}-\left(1-i\sqrt{d}\right)\sqrt{\frac{r(\pi -1)}{1+d}}\left(\Delta \alpha_{s}^{†}+\Delta \alpha_{s}\right)-\sqrt{\frac{1-i\sqrt{d}}{2}}\xi_{C}^{†},$$

(34) $$\frac{d\Delta \alpha_{s}}{dt}=+\left(1-i\sqrt{d}\right)\sqrt{\frac{r(\pi -1)}{1+d}}\left(\Delta \alpha_{p}+\Delta \alpha_{p}^{†}\right)+\sqrt{\frac{1+i\sqrt{d}}{2}\frac{r(\pi -1)}{1+d}}\xi_{C}^{*}+\xi_{C}^{{‡}^{\prime }},$$

(35) $$\frac{d\Delta \alpha_{s}^{†}}{dt}=+\left(1+i\sqrt{d}\right)\sqrt{\frac{r(\pi -1)}{1+d}}\left(\Delta \alpha_{p}+\Delta \alpha_{p}^{†}\right)+\sqrt{\frac{1-i\sqrt{d}}{2}\frac{r(\pi -1)}{1+d}}\xi_{C}^{†*}+\xi_{C}^{{‡}^{\prime }*}.$$

Here, $r=\gamma_{p}/\gamma_{s}$ and $\xi_{C}^{{‡}^{\prime }}=\xi_{C}^{‡}e^{-i\phi }e^{i\sqrt{d}\gamma_{s}t}$. The time *t* is normalized so that $1/\gamma_{s}$ is the unit time. The equivalent equations written with canonical coordinates and momenta are shown in Appendix C.

From these equations in the limit of r≫1, we can calculate the steady-state value of Mandel’s *Q* parameter [56] for a signal mode. It is defined as $Q_{M,s}:=\frac{\langle \Delta {\hat{n}}_{s}^{2}\rangle -\langle {\hat{n}}_{s}\rangle }{\langle {\hat{n}}_{s}\rangle }$, where ${\hat{n}}_{s}={\hat{a}}_{s}^{†}{\hat{a}}_{s}$ and $\Delta {\hat{n}}_{s}={\hat{n}}_{s}-\langle {\hat{n}}_{s}\rangle$. When $Q_{M,s}$ is smaller than zero, the signal mode is in an amplitude squeezed state. From the calculation shown in the Appendix D,

(36) $$Q_{M,s}=-\frac{4+j}{4(2+j)}+\frac{1+d}{4(\pi -1)}-\frac{d}{4\pi }+\frac{\left(1+j)(\pi +d\right)}{\left(2+j\right)[\left(2+j\right)\pi^{2}-2\pi +dj]}$$

is obtained. In the large excitation limit (π→∞), $Q_{M,s}$ converges to a negative value $-\frac{4+j}{4(2+j)}$. Therefore, an amplitude squeezed state is obtained even in a small-*G* NOPO. In the single NOPO limit (j→0), $Q_{M,s}=-\frac{1}{2}+\frac{1+d}{2(\pi -1)}-\frac{d}{2\pi }$. This value converges to $Q_{M,s}\to -\frac{1}{2}$ for a large pump excitation, which is identical to the value obtained in Ref. [35]. In terms of the order O(d), Mandel’s *Q* parameter is written as $Q_{M,s}=-\frac{1}{2}+\frac{1}{2(p-1)}-\frac{d}{4p}$. Therefore, for the same *p*, detuning in the parametric interaction increases the amplitude squeezing. In the strong dissipative coupling limit, i.e., j→∞, the Mandel’s *Q* parameter is halved from that of the single NOPO limit.

The steady-state value of HZ1 entanglement criterion is also obtained for large *r*, as

(37) $$\frac{HZ1}{\langle {\hat{a}}_{s}^{†}{\hat{a}}_{s}\rangle }=\frac{j-2}{4j}-\frac{\pi +d}{4\pi (\pi -1)}-\frac{d(\pi -1)}{j(\pi^{2}+d)}\left[1+\frac{(d+1)\pi }{\left(j+1\right)\pi^{2}-\pi +dj}+\frac{\pi (\pi^{2}-2\pi -d)}{\left(j+2\right)\pi^{2}-2\pi +dj}\right].$$

The detailed derivation is provided in Appendix D. Here, in the large excitation limit (π→∞), HZ1 converges as $HZ1/\langle {\hat{a}}_{s}^{†}{\hat{a}}_{s}\rangle \to \frac{j^{2}-4\left(1+d\right)}{4j(j+2)}$. The coupling coefficient *j* must satisfy $j>2\sqrt{1+d}$ for entanglement far above the threshold. Therefore, detuning *d* is not preferred for satisfying above-threshold entanglement criterion, although d>1 must be satisfied for below-threshold entanglement.

The analytical and numerical Mandel’s *Q* parameter and HZ1 entanglement criterion are compared in Figure 1c,d. Both results are obtained for $J/\gamma_{s}=12$ and d=5 and plotted as the function of normalized excitation *p*. The analytical results assume that $\gamma_{p}/\gamma_{s}\to \infty$ and $G/\gamma_{s}\to 0$. The numerical results were obtained by integrating the positive-*Pc*-number SDEs in Appendix B, for $\gamma_{p}/\gamma_{s}=50$ and $G/\gamma_{s}=10^{-7}$. To obtain steady-state statistics from the positive-*P* calculation, we calculated a single trajectory, starting from $\alpha_{pi}=\alpha_{pi}^{†}=\alpha_{si}=\alpha_{si}^{†}=0\left(i=1,2\right)$ with excitation depending on time as $p\left(t\right)=p\min\left(1,\sqrt{t\gamma_{s}/10^{5}}\right)$, and took the time average from $t\gamma_{s}=10^{5}$ to $t\gamma_{s}=10^{6}$. The theoretical Mandel’s $Q_{M,s}$ parameter from Equation (36) becomes negative at p∼1.7. The numerical $Q_{M,s}$ values were slightly larger than the theoretical values due to the finite $\gamma_{p}/\gamma_{s}$ ratio. When d=0, Equation (36) always gave $Q_{M,s}=0$ at p=2. Therefore, the non-degenerate Kerr effect causes amplitude squeezing for smaller *p*. Moreover, the theoretical HZ1 from Equation (37) becomes positive for p∼2.1. The numerical values are slightly smaller than the analytical results due to the finite $\gamma_{p}/\gamma_{s}$ ratio. Above the threshold, the use of a small $\gamma_{p}/\gamma_{s}$ ratio helps to decrease the degrees of both amplitude squeezing and entanglement. This differs from the below threshold case, where using small $\gamma_{p}/\gamma_{s}$ ratio improves the anti-bunching and entanglement.

### 3.4. Numerical Results

**Figure 2:**
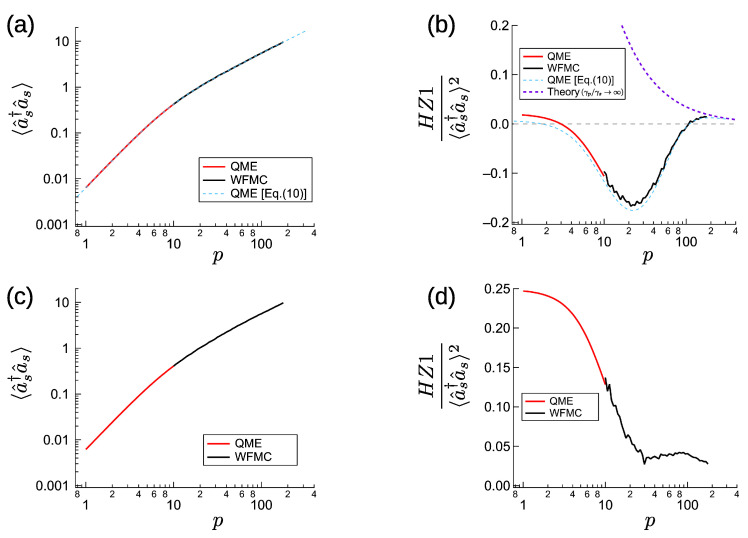
Numerically calculated steady-state of CXM with large pump dissipation, for $\gamma_{p}/\gamma_{s}=50$, $G/\gamma_{s}=400$, d=5. (**a**,**b**) Excitation dependent signal photon number and entanglement criterion for $J/\gamma_{s}=12$. (**c**,**d**) The same results for $J/\gamma_{s}=120$.

Here, we give the numerical simulation results as a function of normalized excitation *p*. The mean signal photon number $\langle {\hat{a}}_{s}^{†}{\hat{a}}_{s}\rangle$ and HZ1 entanglement criterion are presented in Figure 2a,b for $\gamma_{p}/\gamma_{s}=50$, $G/\gamma_{s}=400$, d=5, and $J/\gamma_{s}=12$. For a small excitation *p*, we numerically calculated the quantum master equation (QME) in Equation (3) with a photon number space expansion. When the maximum pump photon number is $M_{p}-1$ and maximum signal photon number is $M_{s}-1$ in the expansion, the size of the density matrix and the calculation time are of order $O(M_{p}^{4}M_{s}^{4})$. This means that the calculation time increases rapidly for large *p*, where the mean signal photon number $\langle {\hat{a}}_{s}^{†}{\hat{a}}_{s}\rangle$ increases and we have to prepare a sufficiently larger $M_{s}$ satisfying $M_{s}\gg \langle {\hat{a}}_{s}^{†}{\hat{a}}_{s}\rangle$. The maximum value of *p* calculated by QME was p=10, where the mean signal photon number is $\langle {\hat{a}}_{s}^{†}{\hat{a}}_{s}\rangle ∼0.42$, and we have used $M_{p}=4$ and $M_{s}=7$. The method of the QME calculation is the same as for Figure 1a,b.

For a larger excitation *p*, we used Mølmer’s wave-function Monte Carlo (WFMC) calculation [42], whose calculation time is $O(M_{p}^{2}M_{s}^{2})$. In the WFMC calculation, time development started from the vacuum state with the excitation $p\left(t\right)=p\min\left(1,\sqrt{t\gamma_{s}/10^{2}}\right)$. A time average was taken from $t\gamma_{s}=10^{2}$ to $t\gamma_{s}=10^{3}-2.5\times 10^{4}$, depending on the values of normalized excitation *p*. The calculation for small *p* with $\langle {\hat{a}}_{s}^{†}{\hat{a}}_{s}\rangle <1$ required more samples (longer period for time averaging) due to the slow convergence, although the size of the Hilbert space is smaller than that of the case of large *p*. The maximum *p* value calculated by WFMC was p∼178, where the mean signal photon number was $\langle {\hat{a}}_{s}^{†}{\hat{a}}_{s}\rangle ∼10$, where we set $M_{p}=4$ and $M_{s}=36$. The blue dashed line shows the results of the density matrix calculation with the pump mode eliminated in Equation (10). The calculation time of this method is $O(M_{s}^{4})$, so we could use it to calculate even the above-threshold characteristics. We calculated the characteristics at p∼316 with $M_{s}=46$, where $\langle {\hat{a}}_{s}^{†}{\hat{a}}_{s}\rangle ∼17$.

The numerical simulation indicated that the mean signal photon number does not show a rapid increase at the threshold. This behavior is known as a thresholdless lasing [57] and is obtained for a large nonlinear saturation coefficient. For an NOPO, different from a Scully–Lamb laser [55], the dependence of the signal photon number on *p* becomes smaller above the threshold. The below-threshold signal photon number is proportional to $p^{2}$ (Equation (13)), and the above-threshold signal photon number is proportional to *p* (Equation (27)). As a consequence of this behavior, a small stimulated emission below the threshold helps to reduce the signal photon number below the $O(p^{2})$ line. The Hillery-Zubairy’s entanglement criterion is satisfied far-below and far-above the threshold. This is an expected from the analytical results (*K* is much larger than *G* below the threshold, while above the threshold $j>2\sqrt{1+d}$ is satisfied). However, the small stimulated emission below the threshold gives a negative correction to $HZ1/{\langle {\hat{a}}_{s}^{†}{\hat{a}}_{s}\rangle }^{2}$. HZ1 turned negative and reached a minimum value near the threshold (p∼19), where the signal photon number $\langle {\hat{a}}_{s}^{†}{\hat{a}}_{s}\rangle$ exceeds one. Far above the threshold $HZ1/{\langle {\hat{a}}_{s}^{†}{\hat{a}}_{s}\rangle }^{2}$ converges to the theoretical values (purple dash line) obtained from dividing Equation (37) by Equation (27). Figure 2c,d presents the numerical simulation results for larger coupling coefficient $J/\gamma_{s}=120$ (other parameters are the same as in Figure 2a,b). Equation (20) leads us to expected that a larger coupling coefficient increases the normalized HZ1 value far below the threshold. Although a stimulated emission contributes negatively to the entanglement criterion, in a similar way to Figure 2b, HZ1 always has a positive value even at the threshold. We thus obtained entanglement in below, above and at the threshold of the CXM, starting from a large pump mode dissipation.

## 4. Large Dissipative Coupling Limit

In the previous section, we showed that achieving entanglement at the threshold is not straightforward. The correction of the small stimulated emission to normalized HZ1 is negative, and the entanglement criterion at the threshold can only be satisfied by increasing $J/\gamma_{s}$ to the same order of $G/\gamma_{s}$. In Figure 1a,b, it can be seen that a large linewidth ratio ($\gamma_{p}/\gamma_{s}$) reduces the anti-bunching and entanglement. The theory for a large pump mode dissipation assumes the coupling coefficient *J* is much smaller than $\gamma_{p},G,K$, which keeps *J* small and prevents quantum correlation between the two signal modes. Here, we consider another limit where *J* is sufficiently larger than the other parameters ($\gamma_{p},\gamma_{s},G,K$).

### 4.1. Far-Below-Threshold Entanglement

First, we will consider the quantum statistics far below the threshold. Appendix A describes the procedure for deriving quantum statistics for a single NOPO far below the threshold. Briefly, this procedure starts from the truncated Fokker-Planck equation (Equation (A3)) in the positive-*P* representation, after the stimulated emission and Kerr non-linearity terms removed (which we write as $\frac{\partial P}{\partial t}{|}_{NOPO,\varepsilon \to 0}$). We integrate the Fokker-Planck equation to obtain the time development equations of mean amplitude products and use them to derive the steady-state photon number and correlation functions. For a CXM, we start from the following Fokker-Planck equation,

(38) $$\begin{array}{ccc} \frac{\partial P}{\partial t} & = & {\left.\frac{\partial P}{\partial t}\right|}_{NOPO1,\varepsilon \to 0}+J\frac{\partial }{\partial \alpha_{s1}}\left(\alpha_{s1}-\alpha_{s2}\right)P+J\frac{\partial }{\partial \alpha_{s1}^{†}}\left(\alpha_{s1}^{†}-\alpha_{s2}^{†}\right)P\\ & + & {\left.\frac{\partial P}{\partial t}\right|}_{NOPO2,\varepsilon \to 0}+J\frac{\partial }{\partial \alpha_{s2}}\left(\alpha_{s2}-\alpha_{s1}\right)P+J\frac{\partial }{\partial \alpha_{s2}^{†}}\left(\alpha_{s2}^{†}-\alpha_{s1}^{†}\right)P. \end{array}$$

Some of the steady-state results obtained from this Fokker-Planck equation are similar to, or even identical to the results for a single NOPO in Appendix A. Even with the large coupling of a signal mode, the pump amplitudes satisfy $\langle \alpha_{p}^{†i}\alpha_{p}^{j}\rangle ={\left(\frac{\gamma_{p}p}{\gamma_{p}+G}\sqrt{\frac{\gamma_{s}}{G}}\right)}^{i+j}$, which is identical to those of a single NOPO. However, as J→∞, the signal photon number becomes one half of that of a single NOPO $\langle \alpha_{s1}^{†}\alpha_{s1}\rangle ∼\frac{\gamma_{p}^{2}p^{2}}{2{\left(\gamma_{p}+G\right)}^{2}}$. This is the same as the J→∞ limit of Equation (13) obtained for large pump mode dissipation, and is also identical to the same limit of the two signal amplitudes’ correlation $\langle \alpha_{s1}^{†}\alpha_{s2}\rangle$ in Equation (14). We assume that such amplitude products connected by *J*, i.e., $\langle \alpha_{p1}^{†i}\alpha_{p1}^{j}\alpha_{s1}^{†k}\alpha_{s1}^{l}\rangle$, to any replacement of $\alpha_{s1}^{†}\to \alpha_{s2}^{†}$ and $\alpha_{s1}\to \alpha_{s2}$, have the same value in the zeroth order of 1/J.

The details of the derivation of $g_{s}^{\left(2\right)}\left(0\right)$ are shown in Appendix E. In the large-*J* limit, the below-threshold second-order correlation function $g_{s}^{\left(2\right)}\left(0\right)$ can be written as,

(39) gs(2)(0)=4γs+2(γp+G)Reγp+2γs+G2γp+4γs+3G+iK2γp+2γs+3G.

In the limit $\gamma_{s}\to 0$, this value converges to $g_{s}^{\left(2\right)}\left(0\right)=\frac{8{\left(\gamma_{p}+G\right)}^{2}}{{\left(2\gamma_{p}+3G\right)}^{2}+K^{2}}$. This is different from the J→∞ limit of Equation (19). If we start from large dissipative coupling J→∞, the anti-bunching state $g_{s}^{\left(2\right)}\left(0\right)\to 0$ is obtained with large non-degenerate Kerr coefficient *K*. From Equation (39), the below-threshold entanglement criterion obeys as $HZ1/{\langle {\hat{a}}_{s}^{†}{\hat{a}}_{s}\rangle }^{2}=1-g_{s}^{\left(2\right)}\left(0\right)$. The normalized HZ1 with no detuning in the parametric interaction (K=0) is

(40) $$\frac{HZ1}{{\langle {\hat{a}}_{s}^{†}{\hat{a}}_{s}\rangle }^{2}}=\frac{G^{2}-2G\left(2\gamma_{p}+5\gamma_{s}\right)-4\left(\gamma_{p}+\gamma_{s}\right)\left(\gamma_{p}+2\gamma_{s}\right)}{\left(2\gamma_{p}+2\gamma_{s}+3G\right)\left(2\gamma_{p}+4\gamma_{s}+3G\right)}.$$

This equation shows that the far-below-threshold entanglement criterion is satisfied even with K=0. When $\gamma_{p}=\gamma_{s}$, it is satisfied for $G/\gamma_{s}>15.6$.

**Figure 3:**
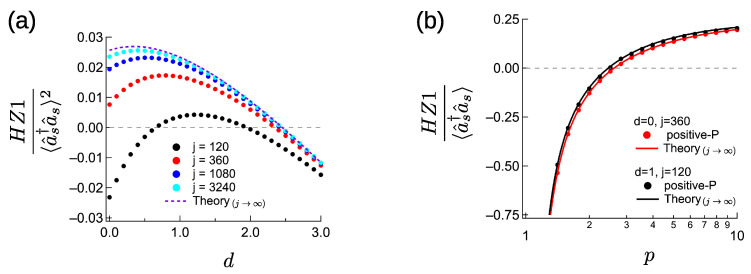
Far-below and far-above threshold characteristics of CXM with large dissipative coupling ($j:=J/\gamma_{s}\gg 1$), for $\gamma_{p}/\gamma_{s}=4$. (**a**) Comparison of far-below threshold theory ($J/\gamma_{s}\to \infty$) and quantum master equation with p=0.01, for $G_{0}/\gamma_{s}=40$. (**b**) Comparison of far-above threshold theory ($J/\gamma_{s}\to \infty$) and positive-*P* calculation with $G/\gamma_{s}=10^{-7}$.

Figure 3a compares the normalized entanglement criterion $HZ1/{\langle {\hat{a}}_{s}^{†}{\hat{a}}_{s}\rangle }^{2}$ obtained from the quantum master Equation (3) and the theory in the J→∞ limit ($1-g_{s}^{\left(2\right)}\left(0\right)$ with Equation (39)). We set the linewidth ratio $\gamma_{p}/\gamma_{s}$ to 4 and the maximum parametric gain $G_{0}/\gamma_{s}=\kappa^{2}/\left(\gamma_{i}\gamma_{s}\right)$ to 40 and investigate the impact of normalized detuning *d* in the parametric interaction. With detuning, the parametric gain becomes $G=\frac{G_{0}}{1+d}$ and the non-degenerate Kerr effect is $K=\frac{G_{0}\sqrt{d}}{1+d}$. The numerical methods are the same as in Figure 1b. The numerical results with the largest *j* (j=3240) have almost the same values as those in the theory assuming J→∞. Even in the J→∞ limit, normalized HZ1 slightly increases for a small detuning *d*. As *d* increases, *K* decreases as $\propto \frac{1}{\sqrt{d}}$ and gets closer to $\gamma_{s}$, where below-threshold entanglement is impossible. When $J/\gamma_{s}=360$, entanglement criterion is satisfied for d=0. However, when $J/\gamma_{s}=120$, the entanglement criterion is not satisfied with zero detuning, although HZ1 becomes positive with a non-zero detuning parameter.

### 4.2. Far-Above-Threshold Entanglement

Next, we derive the far-above-threshold entanglement in the limit J→∞. First of all, we will consider a single NOPO. From the cSDEs in Equations (32) and (33), we obtain the equations of the pump amplitude fluctuations, $\Delta X_{p}=\frac{\Delta \alpha_{p}+\Delta \alpha_{p}^{†}}{\sqrt{2}}$ and $\Delta P_{p}=\frac{\Delta \alpha_{p}-\Delta \alpha_{p}^{†}}{\sqrt{2}i}$. The equations in this representation are shown in Appendix C. The mean fluctuation products are

(41) $$\begin{array}{c} \frac{d\langle \Delta X_{p}^{2}\rangle }{dt}=-2rp\cos\phi \langle \Delta X_{p}^{2}\rangle +2rp\sin\phi \langle \Delta X_{p}\Delta P_{p}\rangle -4\sqrt{\frac{r(\pi -1)}{1+d}}\langle \Delta X_{p}\Delta X_{s}\rangle , \end{array}$$

(42) $$\begin{array}{c} \frac{d\langle \Delta P_{p}^{2}\rangle }{dt}=-2rp\cos\phi \langle \Delta P_{p}^{2}\rangle -2rp\sin\phi \langle \Delta X_{p}\Delta P_{p}\rangle -4\sqrt{\frac{rd(\pi -1)}{1+d}}\langle \Delta P_{p}\Delta X_{s}\rangle , \end{array}$$

(43) $$\begin{array}{ccc} \frac{d\langle \Delta X_{p}\Delta P_{p}\rangle }{dt} & = & -2rp\cos\phi \langle \Delta X_{p}\Delta P_{p}\rangle -rp\sin\phi \langle \Delta X_{p}^{2}\rangle +rp\sin\phi \langle \Delta P_{p}^{2}\rangle \\ & - & 2\sqrt{\frac{r(\pi -1)}{1+d}}\langle \Delta P_{p}\Delta X_{s}\rangle -2\sqrt{\frac{rd(\pi -1)}{1+d}}\langle \Delta X_{p}\Delta X_{s}\rangle . \end{array}$$

These pump amplitude fluctuations are excited by two fluctuation correlations, $\langle \Delta X_{p}\Delta X_{s}\rangle$, and $\langle \Delta P_{p}\Delta X_{s}\rangle$, where $\Delta X_{s}$ is the amplitude fluctuation of the signal mode. We introduce the normalized amplitude correlations,

(44) $$B=-2\sqrt{\frac{r(\pi -1)}{1+d}}\langle \Delta X_{p}\Delta X_{s}\rangle ,$$

(45) $$A=-2\sqrt{\frac{r(\pi -1)}{1+d}}\langle \Delta P_{p}\Delta X_{s}\rangle .$$

The steady-state pump amplitude fluctuations in terms of *A* and *B* are

(46) $$\langle \Delta X_{p}^{2}\rangle =\frac{1+d}{2r(\pi +d)}\left(B+\sqrt{d}A\right)+\frac{\pi B-\sqrt{d}A}{2rp^{2}},$$

(47) $$\langle \Delta P_{p}^{2}\rangle =\frac{1+d}{2r(\pi +d)}\left(B+\sqrt{d}A\right)-\frac{\pi B-\sqrt{d}A}{2rp^{2}},$$

(48) $$2\langle \Delta X_{p}\Delta P_{p}\rangle =\frac{\pi A+\sqrt{d}B}{rp^{2}}.$$

Next, we obtain the time development equations of $\langle \Delta X_{p}\Delta X_{s}\rangle$, $\langle \Delta P_{p}\Delta X_{s}\rangle$ and $\langle \Delta X_{s}^{2}\rangle$.

(49) $$\begin{array}{ccc} \frac{d\langle \Delta X_{p}\Delta X_{s}\rangle }{dt} & = & -rp\cos\phi \langle \Delta X_{p}\Delta X_{s}\rangle +rp\sin\phi \langle \Delta P_{p}\Delta X_{s}\rangle \\ & + & 2\sqrt{\frac{r(\pi -1)}{1+d}}\langle \Delta X_{p}^{2}\rangle -\sqrt{\frac{r(\pi -1)}{1+d}}\left(2\langle \Delta X_{s}^{2}\rangle +1\right), \end{array}$$

(50) $$\begin{array}{ccc} \frac{d\langle \Delta P_{p}\Delta X_{s}\rangle }{dt} & = & -rp\cos\phi \langle \Delta P_{p}\Delta X_{s}\rangle -rp\sin\phi \langle \Delta X_{p}\Delta X_{s}\rangle \\ & + & 2\sqrt{\frac{r(\pi -1)}{1+d}}\langle \Delta X_{p}\Delta P_{p}\rangle -\sqrt{\frac{rd(\pi -1)}{1+d}}\left(2\langle \Delta X_{s}^{2}\rangle +1\right), \end{array}$$

(51) $$\frac{d\langle \Delta X_{s}^{2}\rangle }{dt}=4\sqrt{\frac{r(\pi -1)}{1+d}}\langle \Delta X_{p}\Delta X_{s}\rangle +2.$$

B=1 is obtained from the steady state of Equation (51). Substituting Equations (46) and (48) into Equations (49) and (50), we obtain the steady-state signal amplitude fluctuation,

(52) $$\langle \Delta X_{s}^{2}\rangle =-\frac{1}{4}+\frac{1+d}{4(\pi -1)}-\frac{A\sqrt{d}}{4}+\langle \Delta X_{p}^{2}\rangle .$$

Here,

(53) $$A=\frac{\sqrt{d}}{\pi }\frac{\frac{r}{2(\pi -1)}+\frac{1}{\pi +d}+\frac{\pi -1}{\pi^{2}+d}}{\frac{r}{2(\pi -1)}+\frac{1}{\pi +d}-\frac{\pi -1}{\pi^{2}+d}}.$$

This fluctuation theory for a single NOPO is sufficient for calculating the normalized HZ1 in a CXM in the j≫1 limit. HZ1 above the threshold obeys Equation (6) and includes a contributions from canonical momenta. However, for canonical momenta of signal modes, the fluctuation products of the CXM obey

(54) $$\begin{array}{c} \frac{d\langle \Delta P_{s1}^{2}\rangle }{dt}=-4\sqrt{\frac{rd(\pi -1)}{1+d}}\langle \Delta X_{p1}\Delta P_{s1}\rangle -2\left(j+\delta \right)\langle \Delta P_{s1}^{2}\rangle +2j\langle \Delta P_{s1}\Delta P_{s2}\rangle +2, \end{array}$$

(55) $$\begin{array}{c} \frac{d\langle \Delta P_{s1}\Delta P_{s2}\rangle }{dt}=-4\sqrt{\frac{rd(\pi -1)}{1+d}}\langle \Delta X_{p1}\Delta P_{s2}\rangle -2\left(j+\delta \right)\langle \Delta P_{s1}\Delta P_{s2}\rangle +2j\langle \Delta P_{s1}^{2}\rangle . \end{array}$$

The steady-state difference of these two fluctuation values is

(56) $$\begin{array}{c} \langle \Delta P_{s1}^{2}\rangle -\langle \Delta P_{s1}\Delta P_{s2}\rangle =\frac{1}{2j}-\frac{1}{j}\sqrt{\frac{rd(\pi -1)}{1+d}}\left(\langle \Delta X_{p1}\Delta P_{s1}\rangle -\langle \Delta X_{p1}\Delta P_{s2}\rangle \right), \end{array}$$

which converges to zero in the large *j* limit. The sum $\langle \Delta X_{s1}^{2}\rangle +\langle \Delta X_{s1}\Delta X_{s2}\rangle$ appearing in the HZ1 is the same as $\langle \Delta X_{s}^{2}\rangle$ of a single NOPO shown in Equation (52). Finally, the normalized HZ1 in the J→∞ limit is

(57) $$\begin{array}{c} \frac{HZ1}{\langle {\hat{a}}_{s}^{†}{\hat{a}}_{s}\rangle }=\frac{1}{4}-\frac{1+d}{4(\pi -1)}+\frac{\sqrt{d}A}{4}-\frac{\left(1+d\right)\left(1+\sqrt{d}A\right)}{2r(\pi +d)}-\frac{\pi -\sqrt{d}A}{2rp^{2}}. \end{array}$$

Mandel’s *Q* parameter for the signal mode $Q_{M,s}=2\langle \Delta X_{s1}^{2}\rangle$ is the same as $-HZ1/\langle {\hat{a}}_{s}^{†}{\hat{a}}_{s}\rangle ∼\langle \Delta X_{s1}^{2}\rangle +\langle \Delta X_{s1}\Delta X_{s2}\rangle$ in the J→∞ limit. If we take the r→∞ limit in Equation (57), the normalized entanglement criterion converges to $\frac{HZ1}{\langle {\hat{a}}_{s}^{†}{\hat{a}}_{s}\rangle }=\frac{1}{4}-\frac{\pi +d}{4\pi (\pi -1)}$, which is the same as the j→∞ limit of the theory with a large pump dissipation (Equation (37)). This independence of the order of taking limits does not apply below the threshold. The O(1/r) correction to $HZ1/\langle {\hat{a}}_{s}^{†}{\hat{a}}_{s}\rangle$ is negative: $-\frac{1}{r\pi }-\frac{2d(\pi -1)}{r\pi (\pi^{2}+d)}$. Therefore, above-threshold entanglement is more easily obtained with large *r* (see Figure 1d).

We performed a numerical simulation using a positive-*P* equations in Appendix B. From Figure 3a with $\gamma_{p}/\gamma_{s}=4$, we chose two sets of parameters: (d=0,j=360) and (d=1,j=120). Below-threshold entanglement was achieved with these parameters, when we set a large parametric gain $G_{0}/\gamma_{s}=40$. Here, to check the far-above threshold entanglement, we performed the positive-*P* calculation for $G/\gamma_{s}=10^{-7}$, with the same method as Figure 1d. The results are shown in Figure 3b. The numerical results fit the analytical ones (Equation (57)), which assume the J→∞ limit. For d=1,j=120, the numerical HZ1 values are slightly smaller than the theoretical values, because the correction of O(1/j) reduces HZ1, as shown in Equation (37). Nevertheless, the values of normalized HZ1 for d=1 are larger than for d=0, because, as discussed in relation to Figure 1c, the O(d) correction makes it easier for an above-threshold NOPO to have a non-classical state.

### 4.3. Numerical Results

**Figure 4:**
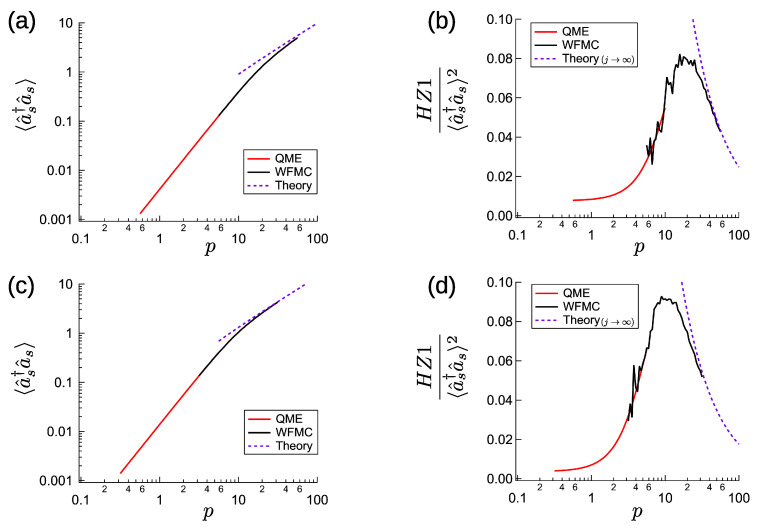
Numerical steady state of CXM with large dissipative coupling ($J/\gamma_{s}\gg 1$), for $G_{0}/\gamma_{s}=40$, $\gamma_{p}/\gamma_{s}=4$. (**a**,**b**) Excitation-dependent signal photon number and entanglement criterion for $d=0,J/\gamma_{s}=360$. (**c**,**d**) The same results for $d=1,J/\gamma_{s}=120$.

Here, we present the numerical simulation results as a function of normalized excitation *p*, for $\gamma_{p}/\gamma_{s}=4,G_{0}/\gamma_{s}=40,d=0$, and j=360. The mean signal photon number and normalized HZ1 are shown in Figure 4a,b. The numerical results are obtained by solving the QME (3) for small-*p*, and by performing a WFMC calculation [42] for large-*p*. The Methods are the same as those used to calculate the results shown in Figure 2, but for WFMC, the time average was taken from $t\gamma_{s}=10^{2}$ to $t\gamma_{s}=10^{4}-10^{5}$ depending on the excitation *p*. For the smallest *p* (∼5.6), time average was taken from $t\gamma_{s}=10^{2}$ to $t\gamma_{s}=10^{5}$ because of the slow convergence. For the maximum p∼56 for WFMC, where the mean signal photon number is $\langle {\hat{a}}_{s}^{†}{\hat{a}}_{s}\rangle ∼5.0$, we used a photon number space with $M_{p}=5$ and $M_{s}=21$ and took the time average from $t\gamma_{s}=10^{2}$ to $t\gamma_{s}=10^{4}$. The purple dashed lines far-above the threshold are plots of Equation (27), or Equation (57) divided by Equation (27). The numerical results converge to the theoretical values far-above the threshold. The entanglement criterion is satisfied below and above the threshold, although the nonlinear Kerr effect is absent (K=0). In contrast to Figure 2b,d, a small stimulated emission gave a positive correction to the normalized entanglement criterion $HZ1/{\langle {\hat{a}}_{s}^{†}{\hat{a}}_{s}\rangle }^{2}$. Because of this correction, the entanglement criterion is satisfied, even at the threshold. Next, we present the numerical simulation results for $\gamma_{p}/\gamma_{s}=4,G_{0}/\gamma_{s}=40,d=1,j=120$. As shown in Figure 4c,d, for the maximum *p* value (∼32) the mean signal photon number was $\langle {\hat{a}}_{s}^{†}{\hat{a}}_{s}\rangle ∼4.3$. We used $M_{p}=6$ and $M_{s}=21$ for calculating this value. As with Figure 4b, stimulated emission gives a positive correction to the normalized HZ1 value, which also results in entanglement at the threshold. As expected from Figure 3b, the peak value of the normalized HZ1 is slightly larger than in Figure 4b.

## 5. Summary

We showed that Hillery-Zubairy’s entanglement criterion is satisfied in coherent XY machines, below, above, and even at the threshold of a CXM consisting of two highly non-Gaussian $\chi^{\left(2\right)}$-NOPOs. We investigated two limits: (1) the pump mode has much larger dissipation than the signal mode, and (2) the dissipative coupling coefficient is much larger than the other parameters. In the first limit, below-threshold entanglement is possible only when the parametric coupling is detuned. In the second limit, below-threshold entanglement was obtained even when the parametric coupling is not detuned. In the first limit, although detuning of the parametric interaction is necessary to achieve below-threshold entanglement, it prevents above-threshold entanglement if *J* is comparable to $\gamma_{s}$. Moreover, the normalized entanglement criterion $HZ1/{\langle {\hat{a}}_{s}^{†}{\hat{a}}_{s}\rangle }^{2}$ is decreased by a small stimulated emission, while in the second limit, the same value increased. The experimentally required $G/\gamma_{s}$ for at-threshold entanglement is smaller for the second limit than the first. From these considerations, the second case with large dissipative coupling seems to be a more effective way to achieve entanglement at the threshold. For a more detailed study, other entanglement criteria should be discussed (In Appendix F, we discussed the Simon’s necessary and sufficient criterion for Gaussian state entanglement [58]). For obtaining entanglement at the threshold, the small stimulated emission correction to below-threshold $HZ1/{\langle {\hat{a}}_{s}^{†}{\hat{a}}_{s}\rangle }^{2}$, would be important. By further increasing *G* and *K* from the values we used in this paper, quantum state production in quantum spin model [59] would be possible in a CXM.

A large parametric interaction, $\kappa /\gamma_{s}∼10^{-2}$, has been experimentally confirmed in a second-order nonlinear ($\chi^{\left(2\right)}$) rib-waveguide-based microring resonator [60]. The theoretical model studied in this paper could be realized by stimulated Raman scattering of traveling-wave modes in a silicon rib-waveguide [61], or standing-wave modes in silicon photonic crystal nanocavities [62,63], coupled via low-*Q* cavity mode [64]. For traveling wave model, the pulse period must be longer than the phonon lifetime to avoid unintentional correlation. In the standing-wave model, the parametric gain normalized by linear dissipation is calculated as $\frac{G}{\gamma_{s}}∼\frac{\hbar c^{2}g_{R}Q_{cs}}{\varepsilon_{r}V_{cav}}∼9\times 10^{-10}Q_{cs}$ [62]. Here, *c* is the speed of light in vaccum, $Q_{cs}$ is the quality factor of the signal cavity mode, $g_{R}$ is the coefficient of Raman amplification, $\varepsilon_{r}$ is the relative dielectric coefficient of the material, and $V_{cav}$ is the volume of the cavity (we used $g_{R}∼57\;\mathrm{cm}/\mathrm{GW}$ [65], $\varepsilon_{r}=12$ and $V_{cav}∼0.5\;m^{3}$ [62] for silicon photonic crystal nanocavity). The cavity quality factor must be as large as $Q_{cs}∼10^{10}$ to achieve below-threshold entanglement. This is only an order of magnitude larger than the numerically achieved *Q* factor [66]. The hyper-parametric oscillation which enabled the CXM with third-order ($\chi^{\left(3\right)}$) nonlinearity [30] can have similar characteristics in terms of below-threshold anti-bunching or above-threshold amplitude squeezing (Appendix G). The CXMs of $\chi^{\left(3\right)}$ NOPOs seem to realize entanglement due to these non-classical characteristics.

## Figures and Tables

**Figure A1:**
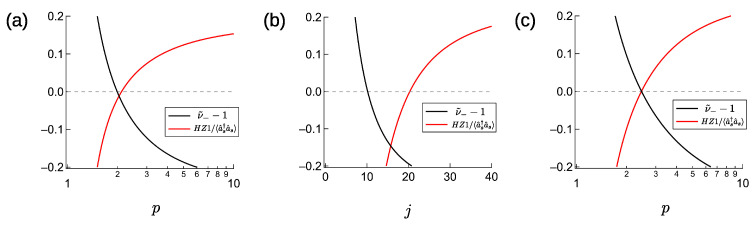
Comparison between Simon’s criterion (${\tilde{\nu }}_{-}-1$) and $HZ1/\langle {\hat{a}}_{s}^{†}{\hat{a}}_{s}\rangle$ for above-threshold CXM. (**a**,**b**) The case of large pump mode dissipation as a function of *p* or *j*. (**c**) The case of large dissipative coupling as a function of *p*.

**Figure A2:**
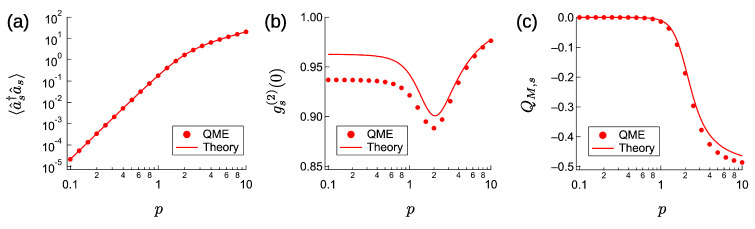
Comparison between QME and analytical results for a hyper-parametric oscillator with $G/\gamma_{s}=\gamma_{p}/\gamma_{s}=100$. (**a**) Mean signal photon number. (**b**) The second order correlation function of the signal mode, $g_{s}^{\left(2\right)}\left(0\right)$. (**c**) Mandel’s *Q* parameter of the signal mode.

## Appendix A. Fokker-Planck Equation and Far-Below Threshold Characteristics of Single NOPO

Here, we derive the below-threshold characteristics of a single NOPO using the positive-*P* Fokker-Planck equation. The positive-*P* distribution function $P(\alpha ,\alpha^{†})$ is defined for a bosonic mode $\hat{a}$ as [40,41],

(A1) $$\hat{\rho }=\int P\left(\alpha ,\alpha^{†}\right)\frac{|\alpha \rangle \langle \alpha^{†*}|}{\langle \alpha^{†*}|\alpha \rangle }d^{2}\alpha d^{2}\alpha^{†}.$$

For a single NOPO obeying Equation (2), the motion of the positive-*P* amplitudes, for the pump mode ($\alpha_{p},\alpha_{p}^{†}$) and for the signal mode ($\alpha_{s},\alpha_{s}^{†}$), depends on the Fokker-Planck equation of the quasi-distribution function $P(\alpha_{p},\alpha_{p}^{†},\alpha_{s},\alpha_{s}^{†})$.

(A2) $$\begin{array}{ccc} \frac{\partial P}{\partial t} & = & \frac{\partial }{\partial \alpha_{p}}\left(\gamma_{p}+G\left(1+\alpha_{s}^{†}\alpha_{s}\right)+iK\alpha_{s}^{†}\alpha_{s}\right)\alpha_{p}P+\frac{\partial }{\partial \alpha_{p}^{†}}\left(\gamma_{p}+G\left(1+\alpha_{s}^{†}\alpha_{s}\right)-iK\alpha_{s}^{†}\alpha_{s}\right)\alpha_{p}^{†}P\\ & + & \frac{\partial }{\partial \alpha_{s}}\left(\gamma_{s}-\left(G-iK\right)\alpha_{p}^{†}\alpha_{p}\right)\alpha_{s}P+\frac{\partial }{\partial \alpha_{s}^{†}}\left(\gamma_{s}-\left(G+iK\right)\alpha_{p}^{†}\alpha_{p}\right)\alpha_{s}^{†}P-\varepsilon \frac{\partial P}{\partial \alpha_{p}}-\varepsilon \frac{\partial P}{\partial \alpha_{p}^{†}}\\ & + & 2G\frac{\partial^{2}}{\partial \alpha_{s}^{†}\partial \alpha_{s}}\left(\alpha_{p}^{†}\alpha_{p}P\right)-\left(G+iK\right)\frac{\partial^{2}}{\partial \alpha_{p}\partial \alpha_{s}}\left(\alpha_{p}\alpha_{s}P\right)-\left(G-iK\right)\frac{\partial^{2}}{\partial \alpha_{p}^{†}\partial \alpha_{s}^{†}}\left(\alpha_{p}^{†}\alpha_{s}^{†}P\right). \end{array}$$

Next, we derive the far below-threshold characteristics (ε→0). Assuming small signal photon number ($\alpha_{s}^{†}\alpha_{s}\ll 1$), we remove terms representing stimulated emission from the pump mode to the signal mode, and terms representing Kerr nonlinearity. After these terms have been removed, the Fokker-Planck equation of $P(\alpha_{p},\alpha_{p}^{†},\alpha_{s},\alpha_{s}^{†})$ becomes

(A3) $$\begin{array}{ccc} {\left.\frac{\partial P}{\partial t}\right|}_{NOPO,\varepsilon \to 0} & = & \left(\gamma_{p}+G\right)\frac{\partial }{\partial \alpha_{p}}\left(\alpha_{p}P\right)+\left(\gamma_{p}+G\right)\frac{\partial }{\partial \alpha_{p}^{†}}\left(\alpha_{p}^{†}P\right)\\ & + & \gamma_{s}\frac{\partial }{\partial \alpha_{s}}\left(\alpha_{s}P\right)+\gamma_{s}\frac{\partial }{\partial \alpha_{s}^{†}}\left(\alpha_{s}^{†}P\right)-\varepsilon \frac{\partial P}{\partial \alpha_{p}}-\varepsilon \frac{\partial P}{\partial \alpha_{p}^{†}}+2G\frac{\partial^{2}}{\partial \alpha_{s}^{†}\partial \alpha_{s}}\left(\alpha_{p}^{†}\alpha_{p}P\right)\\ & - & \left(G+iK\right)\frac{\partial^{2}}{\partial \alpha_{p}\partial \alpha_{s}}\left(\alpha_{p}\alpha_{s}P\right)-\left(G-iK\right)\frac{\partial^{2}}{\partial \alpha_{p}^{†}\partial \alpha_{s}^{†}}\left(\alpha_{p}^{†}\alpha_{s}^{†}P\right). \end{array}$$

From this truncated Fokker-Planck equation, the mean of pump amplitude $\langle \alpha_{p}\rangle$

(=$\int \alpha_{p}Pd^{2}\alpha_{p}d^{2}\alpha_{p}^{†}d^{2}\alpha_{s}d^{2}\alpha_{s}^{†})$ develops as

(A4) $$\frac{d\langle \alpha_{p}\rangle }{dt}=-\left(\gamma_{p}+G\right)\langle \alpha_{p}\rangle +\varepsilon .$$

The steady-state mean pump amplitude is written as

(A5) $$\langle \alpha_{p}\rangle =\frac{\gamma_{p}p}{\gamma_{p}+G}\sqrt{\frac{\gamma_{s}}{G}}.$$

The time-development of higher order moments for the pump mode $\langle \alpha_{p}^{†i}\alpha_{p}^{j}\rangle$ is obtained as,

(A6) $$\frac{d\langle \alpha_{p}^{†i}\alpha_{p}^{j}\rangle }{dt}=-\left(i+j\right)\left(\gamma_{p}+G\right)\langle \alpha_{p}^{†i}\alpha_{p}^{j}\rangle +i\varepsilon \langle \alpha_{p}^{†i-1}\alpha_{p}^{j}\rangle +j\varepsilon \langle \alpha_{p}^{†i}\alpha_{p}^{j-1}\rangle .$$

Here, we neglect terms with a negative exponent in the right hand side. Far below the threshold, these moments in the steady state can be simply written as a product of steady-state mean pump amplitudes $\langle \alpha_{p}^{†i}\alpha_{p}^{j}\rangle ={\langle \alpha_{p}\rangle }^{i+j}$.

Next, the mean signal photon number develops as

(A7) $$\frac{d\langle \alpha_{s}^{†}\alpha_{s}\rangle }{dt}=-2\gamma_{s}\langle \alpha_{s}^{†}\alpha_{s}\rangle +2G\langle \alpha_{p}^{†}\alpha_{p}\rangle .$$

The steady-state signal photon number is written as

(A8) $$\langle \alpha_{s}^{†}\alpha_{s}\rangle =\frac{\gamma_{p}^{2}p^{2}}{{\left(\gamma_{p}+G\right)}^{2}}.$$

Next, we consider higher-order moments containing signal mode amplitudes. To obtain the second-order correlation function of the signal mode $g_{s}^{\left(2\right)}\left(0\right)$, we derive the time development of $\langle \alpha_{s}^{†2}\alpha_{s}^{2}\rangle$ from Equation (A3),

(A9) $$\frac{d\langle \alpha_{s}^{†2}\alpha_{s}^{2}\rangle }{dt}=-4\gamma_{s}\langle \alpha_{s}^{†2}\alpha_{s}^{2}\rangle +8G\langle \alpha_{p}^{†}\alpha_{p}\alpha_{s}^{†}\alpha_{s}\rangle .$$

In the steady-state, the second order correlation function is

(A10) $$g_{s}^{\left(2\right)}\left(0\right)=\frac{\langle \alpha_{s}^{†2}\alpha_{s}^{2}\rangle }{{\langle \alpha_{s}^{†}\alpha_{s}\rangle }^{2}}=2\frac{G\langle \alpha_{p}^{†}\alpha_{p}\alpha_{s}^{†}\alpha_{s}\rangle }{\gamma_{s}{\langle \alpha_{s}^{†}\alpha_{s}\rangle }^{2}}=2g_{X},$$

Here, $g_{X}:=\frac{\langle \alpha_{p}^{†}\alpha_{p}\alpha_{s}^{†}\alpha_{s}\rangle }{\langle \alpha_{p}^{†}\alpha_{p}\rangle \langle \alpha_{s}^{†}\alpha_{s}\rangle }$ is the correlation between the pump photon number and signal photon number. This photon number correlation develops as

(A11) d〈αp†αpαs†αs〉dt=−2(γp+γs+2G)〈αp†αpαs†αs〉+2εRe〈αpαs†αs〉+2G〈αp†2αp2〉.

The steady-state correlation function is

(A12) gX=γs+(γp+G)RegX′γp+γs+2G.

Here, $g_{X}^{\prime }:=\frac{\langle \alpha_{p}\alpha_{s}^{†}\alpha_{s}\rangle }{\langle \alpha_{p}\rangle \langle \alpha_{s}^{†}\alpha_{s}\rangle }$ is the correlation function between pump amplitude and signal photon number. This correlation is written as,

(A13) $$\begin{array}{c} \frac{d\langle \alpha_{p}\alpha_{s}^{†}\alpha_{s}\rangle }{dt}=-\left(\gamma_{p}+2\gamma_{s}+2G+iK\right)\langle \alpha_{p}\alpha_{s}^{†}\alpha_{s}\rangle +\varepsilon \langle \alpha_{s}^{†}\alpha_{s}\rangle +2G\langle \alpha_{p}^{†}\alpha_{p}^{2}\rangle . \end{array}$$

In the steady-state, we achieve.

(A14) $$g_{X}^{\prime }=\frac{\gamma_{s}+2\gamma_{s}+G}{\gamma_{p}+2\gamma_{s}+2G+iK}.$$

The second-order correlation function of the signal mode of a below-threshold single NOPO is written as

(A15) gs(2)(0)=2γs+(γp+G)Reγp+2γs+Gγp+2γs+2G+iKγp+γs+2G.

This value is 2 for small G,K [29], and it converges to zero in the large-*K* limit [33]. Even when K=0 (no detuning in the parametric interaction), a single NOPO can have an anti-bunching state ($g_{s}^{\left(2\right)}\left(0\right)<1$) for large *G*. However, the minimum value is 0.5, instead of zero as obtained from the detuned parametric interaction.

## Appendix B. Positive-P Equations for Numerical Calculation

The Fokker-Planck equation of the positive-*P* distribution function (Equation (A2)) has only first- and second-order derivatives of the positive-*P* amplitudes. Therefore, the Fokker-Planck equation of $P(\alpha_{p},\alpha_{p}^{†},\alpha_{s},\alpha_{s}^{†})$ is rewritten as *c*-number stochastic differential equations (cSDEs) of the complex amplitudes ($\alpha_{p},\alpha_{p}^{†},\alpha_{s},\alpha_{s}^{†}$). For one Fokker-Planck equation, there can be multiple cSDEs with different coefficients of random numbers [41]. We used Equations (21)–(24) to derive the above-threshold fluctuation characteristics. In the numerical calculations to test the above-threshold characteritics, instead, we used the following equations of positive-*P* amplitudes,

(A16) $$\frac{d\alpha_{p}}{dt}=-\left(\gamma_{p}+G\right)\alpha_{p}+\varepsilon -\left(G+iK\right)\alpha_{s}^{†}\alpha_{s}\alpha_{p}-\sqrt{\frac{G+iK}{2}}\alpha_{s}\xi_{C},$$

(A17) $$\frac{d\alpha_{p}^{†}}{dt}=-\left(\gamma_{p}+G\right)\alpha_{p}^{†}+\varepsilon -\left(G-iK\right)\alpha_{s}^{†}\alpha_{s}\alpha_{p}^{†}-\sqrt{\frac{G-iK}{2}}\alpha_{s}^{†}\xi_{C}^{†},$$

(A18) $$\begin{array}{c} \frac{d\alpha_{s}}{dt}=-\gamma_{s}\alpha_{s}+\left(G-iK\right)\alpha_{p}^{†}\alpha_{p}\alpha_{s}+\sqrt{\frac{G+iK}{2}}\alpha_{p}\xi_{C}^{*}+\sqrt{\frac{G-iK}{2}}\alpha_{p}\xi_{C}^{†}, \end{array}$$

(A19) $$\begin{array}{c} \frac{d\alpha_{s}^{†}}{dt}=-\gamma_{s}\alpha_{s}^{†}+\left(G+iK\right)\alpha_{p}^{†}\alpha_{p}\alpha_{s}^{†}+\sqrt{\frac{G-iK}{2}}\alpha_{p}^{†}\xi_{C}^{†*}+\sqrt{\frac{G+iK}{2}}\alpha_{p}^{†}\xi_{C}. \end{array}$$

These equations have two complex random numbers $\left(\xi_{C},\xi_{C}^{†}\right)$ per NOPO, instead of three complex random numbers in Equations (21)–(24). In the CXM consisting of two NOPOs (Equation (3)), the equations of the signal amplitudes have additional terms due to the dissipative coupling Liouvillian, i.e., $\frac{d\alpha_{s1}}{dt}=\frac{d\alpha_{s1}}{dt}{|}_{NOPO1}-J\left(\alpha_{s1}-\alpha_{s2}\right)$, $\frac{d\alpha_{s1}^{†}}{dt}=\frac{d\alpha_{s1}^{†}}{dt}{|}_{NOPO1}-J\left(\alpha_{s1}^{†}-\alpha_{s2}^{†}\right)$, $\frac{d\alpha_{s2}}{dt}=\frac{d\alpha_{s2}}{dt}{|}_{NOPO2}-J\left(\alpha_{s2}-\alpha_{s1}\right)$, and $\frac{d\alpha_{s2}^{†}}{dt}=\frac{d\alpha_{s2}^{†}}{dt}{|}_{NOPO2}-J\left(\alpha_{s2}^{†}-\alpha_{s1}^{†}\right)$. The numerical results of the positive-*P* calculations are shown in Figure 1c,d and Figure 3b. These results are calculated for a small $G/\gamma_{s}$ ratio. However, the positive-*P* calculation becomes unstable for a large $G/\gamma_{s}$ ratio as shown in Figure 2 and Figure 4, although a large $G/\gamma_{s}$ is essential for obtaining below-threshold entanglement. We used QME or WFMC to calculate the characteritics for a large parametric gain.

## Appendix C. On the Fluctuations of Above-Threshold NOPO

Here, we summarize the different representation of above-threshold fluctuations of a single NOPO. After removing the phase factors from Equations (30) and (31), the equations of positive-*P* amplitudes $\Delta \alpha_{k},\Delta \alpha_{k}^{†}\left(k=p,s\right)$ become Equations (32)–(35). Here, we rewrite these equations with $\Delta X_{k}=\frac{\Delta \alpha_{k}+\Delta \alpha_{k}^{†}}{\sqrt{2}}\left(k=p,s\right)$, and $\Delta P_{k}=\frac{\Delta \alpha_{k}-\Delta \alpha_{k}^{†}}{\sqrt{2}i}\left(k=p,s\right)$,

(A20) $$\frac{d\Delta X_{p}}{dt}=-rp\cos\phi \Delta X_{p}+rp\sin\phi \Delta P_{p}-2\sqrt{\frac{r(\pi -1)}{1+d}}\Delta X_{s}-\sqrt{\frac{1+i\sqrt{d}}{4}}\xi_{C}-\sqrt{\frac{1-i\sqrt{d}}{4}}\xi_{C}^{†},$$

(A21) $$\frac{d\Delta P_{p}}{dt}=-rp\cos\phi \Delta P_{p}-rp\sin\phi \Delta X_{p}-2\sqrt{\frac{rd(\pi -1)}{1+d}}\Delta X_{s}+i\sqrt{\frac{1+i\sqrt{d}}{4}}\xi_{C}-i\sqrt{\frac{1-i\sqrt{d}}{4}}\xi_{C}^{†},$$

(A22) $$\frac{d\Delta X_{s}}{dt}=2\sqrt{\frac{r(\pi -1)}{1+d}}\Delta X_{p}+\sqrt{\frac{1+i\sqrt{d}}{4}\frac{r(\pi -1)}{1+d}}\xi_{C}^{*}+\sqrt{\frac{1-i\sqrt{d}}{4}\frac{r(\pi -1)}{1+d}}\xi_{C}^{†*}+\sqrt{2}\xi_{R1},$$

(A23) $$\frac{d\Delta P_{s}}{dt}=-2\sqrt{\frac{rd(\pi -1)}{1+d}}\Delta X_{p}-i\sqrt{\frac{1+i\sqrt{d}}{4}\frac{r(\pi -1)}{1+d}}\xi_{C}^{*}+i\sqrt{\frac{1-i\sqrt{d}}{4}\frac{r(\pi -1)}{1+d}}\xi_{C}^{†*}+\sqrt{2}\xi_{R2}.$$

Here, $\xi_{C}$ is a complex random number satisfying $\langle \xi_{C}^{*}\left(t\right)\xi_{C}\left(t^{\prime }\right)\rangle =2\delta \left(t-t^{\prime }\right)$. $\xi_{Ri}\left(i=1,2\right)$ are real random numbers satisfying $\langle \xi_{Ri}\left(t\right)\xi_{Rj}\left(t^{\prime }\right)\rangle =\delta_{ij}\delta \left(t-t^{\prime }\right)$. ϕ is the phase factor of the pump mode defined in Equation (29). It satisfies $p\cos\phi =\frac{\pi +d}{1+d}$, and $p\sin\phi =\frac{\sqrt{d}\left(\pi -1\right)}{1+d}$. These equations can also be written as $p(\cos\phi +\sqrt{d}\sin\phi )=\pi$, and $p\left(\sqrt{d}\cos\phi -\sin\phi \right)=\sqrt{d}$.

## Appendix D. Details of Above-Threshold Theory in the Large Pump Mode Dissipation Limit

In this appendix, we derive the above-threshold theory of CXM in the large pump mode dissipation limit. Assuming large r(≫1), we can eliminate the pump mode and obtain the equations of signal-mode amplitude fluctuations from Equations (32)–(35), as

(A24) $$\begin{array}{c} \frac{d\Delta \alpha_{s}}{dt}=-2\left(1-i\sqrt{d}\right)\frac{\pi (\pi -1)}{p^{2}\left(1+d\right)}\left(\Delta \alpha_{s}+\Delta \alpha_{s}^{†}\right)+\sqrt{\frac{\pi }{p^{2}}}\xi_{C}+i\sqrt{\frac{2\left(\pi -1\right)\left(\pi +i\sqrt{d}\right)}{p^{2}\left(1+i\sqrt{d}\right)}}\xi_{R}, \end{array}$$

(A25) $$\begin{array}{c} \frac{d\Delta \alpha_{s}^{†}}{dt}=-2\left(1+i\sqrt{d}\right)\frac{\pi (\pi -1)}{p^{2}\left(1+d\right)}\left(\Delta \alpha_{s}+\Delta \alpha_{s}^{†}\right)+\sqrt{\frac{\pi }{p^{2}}}\xi_{C}^{*}-i\sqrt{\frac{2\left(\pi -1\right)\left(\pi -i\sqrt{d}\right)}{p^{2}\left(1-i\sqrt{d}\right)}}\xi_{R}^{†}. \end{array}$$

Here, $\xi_{R}$ and $\xi_{R}^{†}$ are independent real Gaussian noise satisfying $\langle \xi_{R}\left(t\right)\xi_{R}\left(t^{\prime }\right)\rangle =\delta \left(t-t^{\prime }\right)$ and $\langle \xi_{R}^{†}\left(t\right)\xi_{R}^{†}\left(t^{\prime }\right)\rangle =\delta \left(t-t^{\prime }\right)$.

Using these equations, we will consider an CXM consisting of two NOPOs. Defining the coefficients, $A:=2\left(1-i\sqrt{d}\right)\frac{\pi (\pi -1)}{p^{2}\left(1+d\right)}$, $B:=\frac{2\pi }{p^{2}}$, and $D:=-\frac{2\left(\pi -1\right)\left(\pi +i\sqrt{d}\right)}{p^{2}\left(1+i\sqrt{d}\right)}$, the fluctuations of the canonical coordinates obey,

(A26) d〈ΔXs12〉dt=−2(2ReA+j)〈ΔXs12〉+2j〈ΔXs1ΔXs2〉+ReD+B,

(A27) d〈ΔXs1ΔXs2〉dt=−2(2ReA+j)〈ΔXs1ΔXs2〉+2j〈ΔXs12〉,

where $j=J/\gamma_{s}$. Assuming a Gaussian state, the Mandel’s *Q* parameter is obtained as QM,s=2〈ΔXs12〉=ReD+B4(1ReA+1ReA+j) (shown in Equation (36)).

Next, we calculate the above-threshold values of Hillery-Zubairy’s entanglement criterion following Equation (6). The first two terms related to $\Delta X_{s}$ are obtained as 〈ΔXs12〉+〈ΔXs1ΔXs2〉=ReD+B4ReA=−14+1+d4(π−1)−d4π. The mean fluctuation of $\Delta P_{s}$ diverges above the threshold of the NOPO. We can use a small lasing linewidth parameter δ≪1 to produce a finite fluctuation,

(A28) d〈ΔPs12〉dt=−4ImA〈ΔXs1ΔPs1〉−2(j+δ)〈ΔPs12〉+2j〈ΔPs1ΔPs2〉−ReD+B,

(A29) d〈ΔPs1ΔPs2〉dt=−4ImA〈ΔXs1ΔPs2〉−2(j+δ)〈ΔPs1ΔPs2〉+2j〈ΔPs12〉.

The HZ1 contains the difference of these values, which is independent of small δ,

(A30) 〈ΔPs12〉−〈ΔPs1ΔPs2〉=B−ReD−4ImA(〈ΔXs1ΔPs1〉−〈ΔXs1ΔPs2〉)4j.

Next, we consider the mean products of the $\Delta X_{s}$ and $\Delta P_{s}$ amplitudes, appearing in Equation (A30):

(A31) d〈ΔXs1ΔPs1〉dt=−2(ReA+j)〈ΔXs1ΔPs1〉−2ImA〈ΔXs12〉+2j〈ΔXs1ΔPs2〉+ImD,

(A32) d〈ΔXs1ΔPs2〉dt=−2(ReA+j)〈ΔXs1ΔPs2〉−2ImA〈ΔXs1ΔXs2〉+2j〈ΔXs1ΔPs1〉.

Since 〈ΔXs12〉−〈ΔXs1ΔXs2〉=ReD+B4(ReA+j), the steady-state difference of these values is

(A33) 〈ΔXs1ΔPs1〉−〈ΔXs1ΔPs2〉=ImD−2ImAReD+B4(j+ReA)2(ReA+2j).

Finally, the normalized HZ1 criterion is obtained as Equation (37).

## Appendix E. Details of Below-Threshold Theory in the Large Dissipative Coupling Limit

In this Appendix, we derive the second order correlation function $g_{s}^{\left(2\right)}\left(0\right)$ for below-threshold CXM in the large dissipative coupling limit, using the assumption that values coupled by *J* have the same value in the large *J* limit. $\langle \alpha_{s1}^{†2}\alpha_{s1}^{2}\rangle$ and the three values coupled to it via *J* obey,

(A34) d〈αs1†2αs12〉dt=−4(γs+J)〈αs1†2αs12〉+4JRe〈αs1†2αs1αs2〉+8G〈αp1†αp1αs1†αs1〉,

(A35) $$\begin{array}{ccc} \frac{d\langle \alpha_{s1}^{†2}\alpha_{s1}\alpha_{s2}\rangle }{dt} & = & -4\left(\gamma_{s}+J\right)\langle \alpha_{s1}^{†2}\alpha_{s1}\alpha_{s2}\rangle +2J\langle \alpha_{s1}^{†}\alpha_{s1}\alpha_{s2}^{†}\alpha_{s2}\rangle +J\langle \alpha_{s1}^{†2}\alpha_{s1}^{2}\rangle +J\langle \alpha_{s1}^{†2}\alpha_{s2}^{2}\rangle \\ & + & 4G\langle \alpha_{p1}^{†}\alpha_{p1}\alpha_{s1}^{†}\alpha_{s2}\rangle , \end{array}$$

(A36) $$\frac{d\langle \alpha_{s1}^{†2}\alpha_{s2}^{2}\rangle }{dt}=-4\left(\gamma_{s}+J\right)\langle \alpha_{s1}^{†2}\alpha_{s2}^{2}\rangle +4J\langle \alpha_{s1}^{†2}\alpha_{s1}\alpha_{s2}\rangle ,$$

(A37) d〈αs1†αs1αs2†αs2〉dt=−4(γs+J)〈αs1†αs1αs2†αs2〉+4JRe〈αs1†2αs1αs2〉+4G〈αp1†αp1αs2†αs2〉.

There is no coupling coefficient *J* in the time-development equation of 〈αs1†2αs12〉+2〈αs1†αs1αs2†αs2〉+Re〈αs1†2αs22〉+4Re〈αs1†2αs1αs2〉8. Assuming that this weighted average is identical to $\langle \alpha_{s1}^{†2}\alpha_{s1}^{2}\rangle$ in the zeroth order of 1/J, we obtain

(A38) $$\frac{d\langle \alpha_{s1}^{†2}\alpha_{s1}^{2}\rangle }{dt}∼-4\gamma_{s}\langle \alpha_{s1}^{†2}\alpha_{s1}^{2}\rangle +4G\langle \alpha_{p1}^{†}\alpha_{p1}\alpha_{s1}^{†}\alpha_{s1}\rangle .$$

From $\langle \alpha_{s1}^{†}\alpha_{s1}\rangle =\frac{G}{2\gamma_{s}}\langle \alpha_{p1}^{†}\alpha_{p1}\rangle$, the steady-state value of the second-order correlation function can be written as $g_{s}^{\left(2\right)}\left(0\right)=2g_{X}$. Here, $g_{X}=\frac{\langle \alpha_{p1}^{†}\alpha_{p1}\alpha_{s1}^{†}\alpha_{s1}\rangle }{\langle \alpha_{p1}^{†}\alpha_{p1}\rangle \langle \alpha_{s1}^{†}\alpha_{s1}\rangle }$ is the correlation function between the pump photon number and signal photon number.

Next, we calculate the correlation $g_{X}$ between the pump and signal photon numbers from the three following equations:

(A39) d〈αp1†αp1αs1†αs1〉dt=−2(γp+γs+2G+J)〈αp1†αp1αs1†αs1〉+2JRe〈αp1†αp1αs1†αs2〉+2G〈αp1†2αp12〉+2εRe〈αp1αs1†αs1〉,

(A40) $$\begin{array}{ccc} \frac{d\langle \alpha_{p1}^{†}\alpha_{p1}\alpha_{s1}^{†}\alpha_{s2}\rangle }{dt} & = & -2\left(\gamma_{p}+\gamma_{s}+\frac{3}{2}G+J\right)\langle \alpha_{p1}^{†}\alpha_{p1}\alpha_{s1}^{†}\alpha_{s2}\rangle +J\langle \alpha_{p1}^{†}\alpha_{p1}\alpha_{s1}^{†}\alpha_{s1}\rangle \\ & + & J\langle \alpha_{p1}^{†}\alpha_{p1}\alpha_{s2}^{†}\alpha_{s2}\rangle +\varepsilon \langle \alpha_{p1}\left(\alpha_{s1}^{†}\alpha_{s2}+\alpha_{s2}^{†}\alpha_{s1}\right)\rangle , \end{array}$$

(A41) d〈αp1†αp1αs2†αs2〉dt=−2(γp+γs+G+J)〈αp1†αp1αs2†αs2〉+2JRe〈αp1†αp1αs1†αs2〉+2G〈αp1†αp1αp2†αp2〉+2εRe〈αp1αs2†αs2〉.

Moreover, there is no coupling coefficient *J* in the equation of 〈αp1†αp1αs1†αs1〉+2Re〈αp1†αp1αs1†αs2〉+〈αp1†αp1αs2†αs2〉4. Assuming that this equation is identical to $\langle \alpha_{p1}^{†}\alpha_{p1}\alpha_{s1}^{†}\alpha_{s1}\rangle$ in the zeroth order of inverse coupling coefficient 1/J, the mean photon number product is,

(A42) d〈αp1†αp1αs1†αs1〉dt∼−2γp+γs+32G〈αp1†αp1αs1†αs1〉+G〈αp1†2αp12〉+2εRe〈αp1αs1†αs1〉.

From this equation, the steady-state pump-signal photon number correlation can be written as,

(A43) gX=2γs+(γp+G)RegX′2γp+2γs+3G.

Here, we have used $\varepsilon \langle \alpha_{p1}\rangle ∼\left(\gamma_{p}+G\right)\langle \alpha_{p1}^{†}\alpha_{p1}\rangle$. $g_{X}^{\prime }=\frac{\langle \alpha_{p1}\alpha_{s1}^{†}\alpha_{s1}\rangle }{\langle \alpha_{p1}\rangle \langle \alpha_{s1}^{†}\alpha_{s1}\rangle }$ is the correlation between the pump amplitude and the signal photon number.

To obtain the steady-state correlation function $g_{X}^{\prime }$ between the pump amplitude and the signal photon number, we derived the time development equations of four mean amplitude products,

(A44) $$\begin{array}{ccc} \frac{d\langle \alpha_{p1}\alpha_{s1}^{†}\alpha_{s1}\rangle }{dt} & = & -\left(\gamma_{p}+2\gamma_{s}+2G+iK+2J\right)\langle \alpha_{p1}\alpha_{s1}^{†}\alpha_{s1}\rangle +J\langle \alpha_{p1}\alpha_{s1}^{†}\alpha_{s2}\rangle +J\langle \alpha_{p1}\alpha_{s2}^{†}\alpha_{s1}\rangle \\ & + & 2G\langle \alpha_{p1}^{†}\alpha_{p1}^{2}\rangle +\varepsilon \langle \alpha_{s1}^{†}\alpha_{s1}\rangle , \end{array}$$

(A45) $$\begin{array}{ccc} \frac{d\langle \alpha_{p1}\alpha_{s2}^{†}\alpha_{s2}\rangle }{dt} & = & -\left(\gamma_{p}+2\gamma_{s}+G+2J\right)\langle \alpha_{p1}\alpha_{s2}^{†}\alpha_{s2}\rangle +J\langle \alpha_{p1}\alpha_{s1}^{†}\alpha_{s2}\rangle +J\langle \alpha_{p1}\alpha_{s2}^{†}\alpha_{s1}\rangle \\ & + & 2G\langle \alpha_{p1}\alpha_{p2}^{†}\alpha_{p2}\rangle +\varepsilon \langle \alpha_{s2}^{†}\alpha_{s2}\rangle , \end{array}$$

(A46) $$\frac{d\langle \alpha_{p1}\alpha_{s1}^{†}\alpha_{s2}\rangle }{dt}=-\left(\gamma_{p}+2\gamma_{s}+G+2J\right)\langle \alpha_{p1}\alpha_{s1}^{†}\alpha_{s2}\rangle +J\langle \alpha_{p1}\alpha_{s1}^{†}\alpha_{s1}\rangle +J\langle \alpha_{p1}\alpha_{s2}^{†}\alpha_{s2}\rangle +\varepsilon \langle \alpha_{s1}^{†}\alpha_{s2}\rangle ,$$

(A47) $$\begin{array}{ccc} \frac{d\langle \alpha_{p1}\alpha_{s2}^{†}\alpha_{s1}\rangle }{dt} & = & -\left(\gamma_{p}+2\gamma_{s}+2G+iK+2J\right)\langle \alpha_{p1}\alpha_{s2}^{†}\alpha_{s1}\rangle +J\langle \alpha_{p1}\alpha_{s1}^{†}\alpha_{s1}\rangle +J\langle \alpha_{p1}\alpha_{s2}^{†}\alpha_{s2}\rangle \\ & + & \varepsilon \langle \alpha_{s2}^{†}\alpha_{s1}\rangle . \end{array}$$

The average of these four mean amplitude products obeys the equation without the coupling coefficient *J*. If we assume this average is identical to $\langle \alpha_{p1}\alpha_{s1}^{†}\alpha_{s1}\rangle$ in the zeroth order of 1/J, we obtain

(A48) $$\begin{array}{c} \frac{d\langle \alpha_{p1}\alpha_{s1}^{†}\alpha_{s1}\rangle }{dt}∼-\left(\gamma_{p}+2\gamma_{s}+\frac{3}{2}G+i\frac{K}{2}\right)\langle \alpha_{p1}\alpha_{s1}^{†}\alpha_{s1}\rangle +G\langle \alpha_{p1}^{†}\alpha_{p1}^{2}\rangle +\varepsilon \langle \alpha_{s1}^{†}\alpha_{s1}\rangle . \end{array}$$

From this equation, the steady-state correlation $g_{X}^{\prime }$ between the pump amplitude and the signal photon number is

(A49) $$g_{X}^{\prime }=2\frac{\gamma_{p}+2\gamma_{s}+G}{2\gamma_{p}+4\gamma_{s}+3G+iK}.$$

The second order correlation function $g_{s}^{\left(2\right)}\left(0\right)$ of the far below-threshold large-*J* CXM is obtained in the form of Equation (39).

## Appendix F. Simon’s Criterion for Above-Threshold Entanglement

For entanglement above the threshold, we can use Simon’s criterion [58], which is necessary and sufficient condition for entanglement when the states are Gaussian. When the above-threshold states of CXMs are treated as the Gaussian states with infinite fluctuations of canonical momenta, we can use this criterion. This criterion is described by a covariance matrix of two signal modes $\sigma_{ij}=\langle R_{i}R_{j}\rangle -\langle R_{i}\rangle \langle R_{j}\rangle +\delta_{ij}$, where $\vec{R}=\sqrt{2}\left[X_{s1},P_{s1},X_{s2},P_{s2}\right]$ ($X_{si}$ and $P_{si}\left(i=1,2\right)$ are defined for positive-*P* amplitudes). If the minimum eigenvalue of $i\Omega (\Lambda \sigma \Lambda^{T})$ (which we call ${\tilde{\nu }}_{-}$) is smaller than 1, for a partial-transposition matrix Λ=diag(1,1,1,−1), and $\Omega =\left[\begin{array}{cc} 0 & 1\\ -1 & 0 \end{array}\right]⊕\left[\begin{array}{cc} 0 & 1\\ -1 & 0 \end{array}\right]$, the two signal modes are entangled [67].

From the equations in Appendix C (for $d\Delta P_{s}/dt$, we add $-\delta \Delta P_{s}$ to the right hand side to avoid divergence), we obtain the steady-state values of 20 fluctuation products, $\langle \Delta X_{p1}^{2}\rangle$, $\langle \Delta P_{p1}^{2}\rangle$, $\langle \Delta X_{s1}^{2}\rangle$, $\langle \Delta P_{s1}^{2}\rangle$, $\langle \Delta X_{p1}\Delta P_{p1}\rangle$, $\langle \Delta X_{p1}\Delta X_{s1}\rangle$, $\langle \Delta X_{p1}\Delta P_{s1}\rangle$, $\langle \Delta P_{p1}\Delta X_{s1}\rangle$, $\langle \Delta P_{p1}\Delta P_{s1}\rangle$, $\langle \Delta X_{s1}\Delta P_{s1}\rangle$, and these between different NOPOs. From six values $\langle \Delta X_{s1}^{2}\rangle$, $\langle \Delta P_{s1}^{2}\rangle$, $\langle \Delta X_{s1}\Delta P_{s1}\rangle$, $\langle \Delta X_{s1}\Delta X_{s2}\rangle$, $\langle \Delta P_{s1}\Delta P_{s2}\rangle$, and $\langle \Delta X_{s1}\Delta P_{s2}\rangle$, we calculated the minimum symplectic eigenvalue ${\tilde{\nu }}_{-}$ of a partially transposed covariance matrix.

First, we study the limit of large pump mode dissipation. For $\gamma_{p}/\gamma_{s}=10^{3}$, d=5, j=12, and $\delta =10^{-10}$, we plot the ${\tilde{\nu }}_{-}-1$ as a function of excitation *p*. The comparison with HZ1 criterion is shown in Figure A1a. The entanglement criterion is satisfied for smaller p(∼1.99) than the HZ1 criterion (p∼2.11). In the special case of d=0, the Simon’s criterion is calculated as ${\tilde{\nu }}_{-}^{2}=1-\frac{(j-1)p-2j}{2j(p-1)}$. Therefore, we need j>1 for entanglement in the p→∞ limit, instead of larger j(>2) for HZ1. When $\gamma_{p}/\gamma_{s}=10^{3}$, p=100 and d=100, we show the *j* dependency of ${\tilde{\nu }}_{-}-1$ and the normalized HZ1 in Figure A1b. The required *j* for above-threshold entanglement was also by a factor of 2 smaller for Simon’s criterion. Next, we show the results for large dissipative coupling. For $\gamma_{p}/\gamma_{s}=4$, d=1, $j=10^{3}$, and $\delta =10^{-10}$, we plot the ${\tilde{\nu }}_{-}-1$ in Figure A1c. The Simon’s entanglement criterion is satisfied at the same p(∼2.46) as the HZ1 criterion.

## Appendix G. Quantum Characteristics of Hyper-Parametric Oscillation

In this Appendix, we discuss the steady-state quantum statistics of hyper-parametric oscillation [30] in the limit of large idler mode dissipation and large parametric interaction. In this system, two degenerate pump photons are converted to a signal photon and an idler photon via a $\chi^{\left(3\right)}$ interaction. We use the Liouvillian derived for hyper-Raman scattering [68] with zero-temperature phonons. In this model of hyper-parametric oscillation, the density operator $\hat{\rho }$ follows:

(A50) $$\begin{array}{c} \frac{\partial \hat{\rho }}{\partial t}=\sum_{a=p,s}\gamma_{a}\left(\left[{\hat{a}}_{a},\hat{\rho }{\hat{a}}_{a}^{†}\right]+h.c.\right)+\varepsilon \left[{\hat{a}}_{p}^{†}-{\hat{a}}_{p},\hat{\rho }\right]+G\left(\left[{\hat{a}}_{s}^{†}{\hat{a}}_{p}^{2},\hat{\rho }{\hat{a}}_{p}^{†2}{\hat{a}}_{s}\right]+h.c.\right). \end{array}$$

Here, the last term of Equation (2) are modified and K=0 was assumed. The excitation at the threshold of a hyper-parametric oscillation is written as $p=\varepsilon /\varepsilon_{thr}$, $\varepsilon_{thr}=\gamma_{p}{\left(\gamma_{s}/G\right)}^{1/4}$. As done in Equation (7), we expand the density operator with the complex-*P* representation for the pump mode and the photon number states representation for the signal mode: $\hat{\rho }=\sum_{N_{s}=0}^{\infty }\int P_{N_{s}}\left(\alpha_{p},\alpha_{p}^{†}\right)\frac{|\alpha_{p}\rangle \langle \alpha_{p}^{†*}|}{\langle \alpha_{p}^{†*}|\alpha_{p}\rangle }⊗|N_{s}\rangle \langle N_{s}|d\alpha_{p}d\alpha_{p}^{†}$. We can obtain the time development equation for $P_{N_{s}}\left(\alpha_{p},\alpha_{p}^{†}\right)$,

(A51) $$\begin{array}{ccc} \frac{\partial P_{N_{s}}}{\partial t} & = & 2\gamma_{s}\left(\left(1+N_{s}\right)P_{N_{s}+1}-N_{s}P_{N_{s}}\right)-\varepsilon \frac{\partial P_{N_{s}}}{\partial \alpha_{p}}-\varepsilon \frac{\partial P_{N_{s}}}{\partial \alpha_{p}^{†}}\\ & + & \gamma_{p}\frac{\partial }{\partial \alpha_{p}}\alpha_{p}P_{N_{s}}+2G\left(1+N_{s}\right)\frac{\partial }{\partial \alpha_{p}}\alpha_{p}^{†}\alpha_{p}^{2}P_{N_{s}}-G\left(1+N_{s}\right)\frac{\partial^{2}}{\partial \alpha_{p}^{2}}\alpha_{p}^{2}P_{N_{s}}\\ & + & \gamma_{p}\frac{\partial }{\partial \alpha_{p}^{†}}\alpha_{p}^{†}P_{N_{s}}+2G\left(1+N_{s}\right)\frac{\partial }{\partial \alpha_{p}^{†}}\alpha_{p}^{†2}\alpha_{p}P_{N_{s}}-G\left(1+N_{s}\right)\frac{\partial^{2}}{\partial \alpha_{p}^{†2}}\alpha_{p}^{†2}P_{N_{s}}\\ & + & 2G\alpha_{p}^{†2}\alpha_{p}^{2}\left(N_{s}P_{N_{s}-1}-\left(1+N_{s}\right)P_{N_{s}}\right). \end{array}$$

When the signal photon number is fixed to $N_{s}$, the pump mode has a coherent excitation, linear dissipation depending on $\gamma_{p}$, and two photon absorption depending on $2G(1+N_{s})$. When $G,\gamma_{p}\gg \gamma_{s}$, the pump mode at each $N_{s}$ converges to a steady-state of the system of coherent excitation, linear dissipation, and two-photon absorption. The steady-state complex-*P* distribution function of such a system was derived in Ref. [40], $P_{ss}\left(\alpha_{p},\alpha_{p}^{†},N_{s}\right)∼{\left(\alpha_{p}^{†}\alpha_{p}\right)}^{c-2}e^{2\alpha_{p}^{†}\alpha_{p}+\frac{x}{\alpha_{p}}+\frac{x}{\alpha_{p}^{†}}}$. Here, $x=\frac{\varepsilon }{G(1+N_{s})}$ and $c=\frac{\gamma_{p}}{G(1+N_{s})}$. We write the distribution function as $P_{N_{s}}\left(\alpha_{p},\alpha_{p}^{†}\right)=\rho_{N_{s}}P_{ss}\left(\alpha_{p},\alpha_{p}^{†},N_{s}\right)$. The hopping rate from $N_{s}$ to $N_{s}+1$ is proportional to $\langle \alpha_{p}^{†2}\alpha_{p}^{2}\rangle \left(N_{s}\right)=\int \alpha_{p}^{†2}\alpha_{p}^{2}P_{ss}\left(\alpha_{p},\alpha_{p}^{†},N_{s}\right)d\alpha_{p}d\alpha_{p}^{†}$, which is written as [40]

(A52) $$\langle \alpha_{p}^{†2}\alpha_{p}^{2}\rangle \left(N_{s}\right)=x^{4}\frac{\Gamma {\left(c\right)}^{2}}{\Gamma {\left(c+2\right)}^{2}}\frac{{}_{0}F_{2}\left(c+2,c+2,2x^{2}\right)}{{}_{0}F_{2}\left(c,c,2x^{2}\right)}.$$

From the recursion relation of $\rho_{N_{s}}$, the distribution of signal photon number $\rho_{N_{s}}$ is obtained as

(A53) $$\frac{\rho_{N_{s}+1}}{\rho_{N_{s}}}=\frac{G}{\gamma_{s}}\langle \alpha_{p}^{†2}\alpha_{p}^{2}\rangle \left(N_{s}\right).$$

For $G=\gamma_{p}$, and $G/\gamma_{s}=100$, we show the comparison between numerical results of QME (calculated in the same methods as the main text) and the analytical results obtained by Equations (A52) and (A53). In Figure A2, two methods produced the same mean signal photon number, and the similar behavior of second order correlation function $g_{s}^{\left(2\right)}\left(0\right)$ and Mandel’s *Q* parameter $Q_{M,s}$. Both methods show that the signal mode has a photon anti-bunching state below the threshold and an amplitude squeezed state above the threshold. In this special limit, $g_{s}^{\left(2\right)}\left(0\right)∼\frac{2{\left(\gamma_{p}+G\right)}^{2}}{{\left(\gamma_{p}+2G\right)}^{2}}$ is obtained far below the threshold, which is the same as $\chi^{\left(2\right)}$-NOPO with $K=0,\gamma_{s}\to 0$ (Equation (A15)). For the direct calculation of QME (Equation (A50)), the signal mode has a slightly smaller $g_{s}^{\left(2\right)}\left(0\right)$ and $Q_{M,s}$, due to the finite $\gamma_{p}/\gamma_{s}$ and $G/\gamma_{s}$.
